# Population-specific genetic modification of Huntington's disease in Venezuela

**DOI:** 10.1371/journal.pgen.1007274

**Published:** 2018-05-11

**Authors:** Michael J. Chao, Kyung-Hee Kim, Jun Wan Shin, Diane Lucente, Vanessa C. Wheeler, Hong Li, Jared C. Roach, Leroy Hood, Nancy S. Wexler, Laura B. Jardim, Peter Holmans, Lesley Jones, Michael Orth, Seung Kwak, Marcy E. MacDonald, James F. Gusella, Jong-Min Lee

**Affiliations:** 1 Molecular Neurogenetics Unit, Center for Genomic Medicine, Massachusetts General Hospital, Boston, Massachusetts, United States of America; 2 Department of Neurology, Harvard Medical School, Boston, Massachusetts, United States of America; 3 Institute for Systems Biology, Seattle, Washington, United States of America; 4 Columbia University, New York, New York, United States of America; 5 Departamento de Medicina Interna, Universidade Federal do Rio Grande do Sul, Porto Alegre, Brazil; 6 Serviço de Genética Médica and Laboratório de Identificação Humana, Hospital de Clinicas de Porto Alegre, Porto Alegre, Brazil; 7 Medical Research Council Centre for Neuropsychiatric Genetics and Genomics, Department of Psychological Medicine and Neurology, School of Medicine, Cardiff University, Cardiff, United Kingdom; 8 Department of Neurology, University of Ulm, Ulm, Germany; 9 CHDI Foundation, Princeton, New Jersey, United States of America; 10 Medical and Population Genetics Program, the Broad Institute of M.I.T. and Harvard, Cambridge, Massachusetts, United States of America; 11 Department of Genetics, Harvard Medical School, Boston, Massachusetts, United States of America; University of Minnesota, UNITED STATES

## Abstract

Modifiers of Mendelian disorders can provide insights into disease mechanisms and guide therapeutic strategies. A recent genome-wide association (GWA) study discovered genetic modifiers of Huntington's disease (HD) onset in Europeans. Here, we performed whole genome sequencing and GWA analysis of a Venezuelan HD cluster whose families were crucial for the original mapping of the HD gene defect. The Venezuelan HD subjects develop motor symptoms earlier than their European counterparts, implying the potential for population-specific modifiers. The main Venezuelan HD family inherits *HTT* haplotype hap.03, which differs subtly at the sequence level from European HD hap.03, suggesting a different ancestral origin but not explaining the earlier age at onset in these Venezuelans. GWA analysis of the Venezuelan HD cluster suggests both population-specific and population-shared genetic modifiers. Genome-wide significant signals at 7p21.2–21.1 and suggestive association signals at 4p14 and 17q21.2 are evident only in Venezuelan HD, but genome-wide significant association signals at the established European chromosome 15 modifier locus are improved when Venezuelan HD data are included in the meta-analysis. Venezuelan-specific association signals on chromosome 7 center on *SOSTDC1*, which encodes a bone morphogenetic protein antagonist. The corresponding SNPs are associated with reduced expression of *SOSTDC1* in non-Venezuelan tissue samples, suggesting that interaction of reduced *SOSTDC1* expression with a population-specific genetic or environmental factor may be responsible for modification of HD onset in Venezuela. Detection of population-specific modification in Venezuelan HD supports the value of distinct disease populations in revealing novel aspects of a disease and population-relevant therapeutic strategies.

## Author summary

Huntington’s disease (HD) is a dominantly inherited neurodegenerative disorder caused by a CAG trinucleotide expansion in *HTT*, which encodes huntingtin. HD involves various neurological manifestations such as involuntary movements, cognitive decline, and personality change; age at onset of diagnostic signs is partly determined by the length of the CAG repeat. However, genome-wide analysis of European HD subjects revealed that age at onset corrected for individual CAG repeat size is modified by other genetic factors, revealing potential disease-delaying pathways that form targets for therapeutic strategies. In this study, we set out to identify genetic modifiers in a Venezuelan HD cluster whose families were crucial for discovering the cause of HD. Through genome-wide association analysis using age at onset corrected for CAG repeat size as the phenotype, we found genome-wide significant (chromosome 7) and suggestive significant association signals (chromosomes 4 and 17). Significant modification signals in Venezuelan HD appear to be population-specific because genetic analysis of European HD subjects did not reveal modification signals at those locations. Nevertheless, established modification signals in European HD at a chromosome 15 locus were also augmented by Venezuelan HD data, supporting the existence of both shared and population-specific genetic modifiers in HD. Together with the full sequence of *HTT* shared by the majority of these Venezuelan HD subjects, our genetic analysis provides insights into the geographical origin of the founder mutation, meaningful allele-specific sites for gene targeting approaches, and potential targets for disease-modifying intervention in this population.

## Introduction

Huntington's disease (HD; MIM 143100), a familial neurodegenerative disorder characterized by progressive movement disorder, cognitive decline, and psychiatric disturbances, is caused by an expanded CAG glutamine codon repeat in *HTT*, which encodes huntingtin [1–3]. This genetic defect was originally mapped to chromosome 4p16.3 using linkage analysis in part in Venezuelan families from an HD population cluster whose generous participation in this fundamental HD genetic research also contributed, along with many North American and European families also carrying a CAG expansion mutation, to the discovery of *HTT* [3–6]. The *HTT* repeat is polymorphic in the normal population [7], but inheritance of >35 CAGs can lead to HD, while repeats of >40 CAGs are fully penetrant within a normal lifespan [8,9]. Expanded repeats show frequent germline instability so that HD individuals may not have a CAG repeat length identical to their transmitting parent [10–12]. Rare *de novo* cases of HD are generated sporadically in the population by intergenerational expansion of a normal length CAG repeat [13,14]. The length of the inherited *HTT* CAG repeat is the primary determinant of the age at onset of diagnostic neurological signs, accounting for ~65% of the variance in this phenotype, with longer repeats leading on average to earlier clinical manifestations [7,15–18]. Individuals with biallelic HD mutations (i.e., HD homozygotes) have been reported and support complete dominance with respect to the pathogenic mechanism leading to disease manifestations since the age at onset of an individual with two expanded *HTT* CAG repeats is comparable to that of a heterozygote with the longer of the two repeats [7,19]. Interestingly, based upon one report, despite an age at onset similar to HD heterozygotes, individuals with two expanded *HTT* CAG repeats may display more rapid decline in functional capacity [20]. Although the timing of disease onset is CAG repeat size-dependent, the duration of manifest disease (i.e., the length of time from onset to death) is not [21], suggesting that progression to death involves different tissues or mechanisms than those leading to clinical onset. The relationship between CAG repeat size and HD clinical onset has played an important role in guiding 1) the generation of animal models [22–26], 2) design/interpretation of molecular studies [27], and 3) identification of genetic modifiers [28].

The extensive accumulated experimental data and complete dominance of the mutant allele are most consistent with a gain-of-function mechanism, which has prompted exploration of mutant allele-specific interference with *HTT* expression as a treatment option [29]. Although highly promising in model systems, gene-targeting approaches are just now beginning human trials of safety and efficacy. An alternative strategy for the identification of therapeutic targets is to capitalize on observations from humans with HD to discover naturally occurring modifiers of disease. Although CAG length accounts for a significant proportion of the variance in HD age at onset [15,16,18], each CAG repeat size in the HD population is associated with a wide range of onset ages [7]. Since individual differences between the observed age at clinical onset and the age at clinical onset expected based upon the size of the mutation (i.e., residual age at onset) are partially heritable [30,31], both genetic and environmental factors are thought to influence the timing of onset in addition to CAG length [32]. The HD-associated CAG expansion sits on diverse haplotypes [33–38], reflecting multiple independent mutation events on different polymorphic chromosome backbones. However, none of the most frequent disease chromosome haplotypes is associated with a difference in age at onset [34], arguing that HD is not commonly modified by a common *cis* genetic factor at *HTT*. Similarly, age at diagnostic motor onset of HD is not influenced by the length of the normal CAG repeat [7,32], or by the presence of a second mutant allele [7], suggesting that heritable variance in age at onset for a given CAG repeat is largely due to unlinked *trans* factors [39]. To identify these, we performed an initial genome-wide association (GWA) analysis in HD subjects of European descent that revealed the first set of genome-wide significant loci associated with residual age at onset of motor symptoms. Briefly, there were two independent onset modification signals on chromosome 15 [28], one modification signal on chromosome 8 [28], and one modification signal on chromosome 3 [40]. Two of the modifier loci acted to delay onset and two acted to hasten onset [28,40,41]. These findings established the proof-of-principle that the pathogenesis of HD can be altered prior to emergence of clinical disease. Consistent with single SNP analysis results, pathway analysis supported a role for DNA repair/maintenance pathways in modifying the age at onset of HD, suggesting that somatic size changes of the CAG repeat may play a role in modification of the HD pathogenic process that leads to diagnostic clinical signs [28]. Together, these observations demonstrated that genetic factors in humans are capable of modifying the rate of HD pathogenesis prior to diagnosis and point to an approach based upon such human observations for therapeutic targeting to delay HD onset.

Most genome-wide analyses to date have focused on Europeans, providing important but potentially limited insights into the genetic contribution to disease risk and normal traits (GWAS Catalog; https://www.ebi.ac.uk/gwas/). Our initial European HD GWA study [28] was highly successful in revealing significant modifier loci of relatively strong effect based upon a smaller sample size than most common disease genetic risk studies (GWAS Catalog; https://www.ebi.ac.uk/gwas/). However, we might have missed genetic modifiers not present in Europeans. Here, we have extended our genetic analysis to the Venezuelan HD cluster of non-European HD subjects to: 1) determine the full sequence of *HTT* on the chromosome bearing the CAG expansion mutation; 2) to investigate its origin, and 3) to determine whether modification of age at onset in this Venezuelan population is associated with specific naturally occurring genetic factors.

## Results

### Venezuelan HD subjects

From 1979 to 1999, an interdisciplinary team of investigators annually visited Venezuela to solicit the participation of a large cluster of HD families in research, to document their disease and to obtain blood samples for DNA analysis and preparation of lymphoblastoid cell lines, which were banked for future studies. These HD families contributed to both the localization of the HD defect to chromosome 4 and to discovery of *HTT* and its expanded CAG repeat mutation [3–6]. In 2004, a publication summarizing more than two decades of work by the U.S.–Venezuela Collaborative Research Project described this HD cluster and presented evidence for both genetic and environmental modifiers of HD [30]. For the current study, we prepared DNA from lymphoblastoid cell lines banked prior to 1999 that had relevant clinical and/or relationship information recorded. In all, we genotyped 443 HD and 106 related non-HD Venezuelan subjects using the Illumina Omni2.5 array platform [28] and performed genome-wide imputation using the Michigan Imputation Server (https://imputationserver.sph.umich.edu/index.html) with 1000 Genomes Project data (mixed population) as the reference panel. After quality control (QC) analysis, 374 HD subjects carrying adult onset HD CAG repeat sizes (CAG 40–55) with recorded age at onset of motor symptoms were analyzed in this study. Ancestry of subjects was characterized by comparing to 1000 Genomes Project data (S1 Fig; open black squares represent Venezuelan study subjects), which confirmed the strongest similarity with Columbians (pink square) and Puerto Ricans (pink cross). In addition, familial relationships were inferred based upon estimated IBD from the genome-wide genotype data (S2 Fig), revealing that subjects were from 22 families of quite variable size (S3 Fig). The largest family, arbitrarily termed Family 1, comprised 218 HD individuals with the HD mutation on hap.03, which matches the third most frequent disease chromosome in European HD subjects (S3 Fig) [34,38]. Many of the smaller families (e.g., Family 6 and Family 12) also carry hap.03 and appear to be distantly related to members of the main family, consistent with pedigree reconstruction by the U.S.-Venezuela Collaborative Research Project (S3 Fig). However, the expanded *HTT* CAG repeats in some smaller families occur on different *HTT* haplotypes, including hap.01 (the most frequent European HD haplotype), hap.02, hap.06, hap.07 and hap.12 [34,38], indicating that most but not all HD chromosomes in the Venezuelan cluster are ancestrally related.

### Earlier age at onset in Venezuelan HD subjects

**Fig 1:**
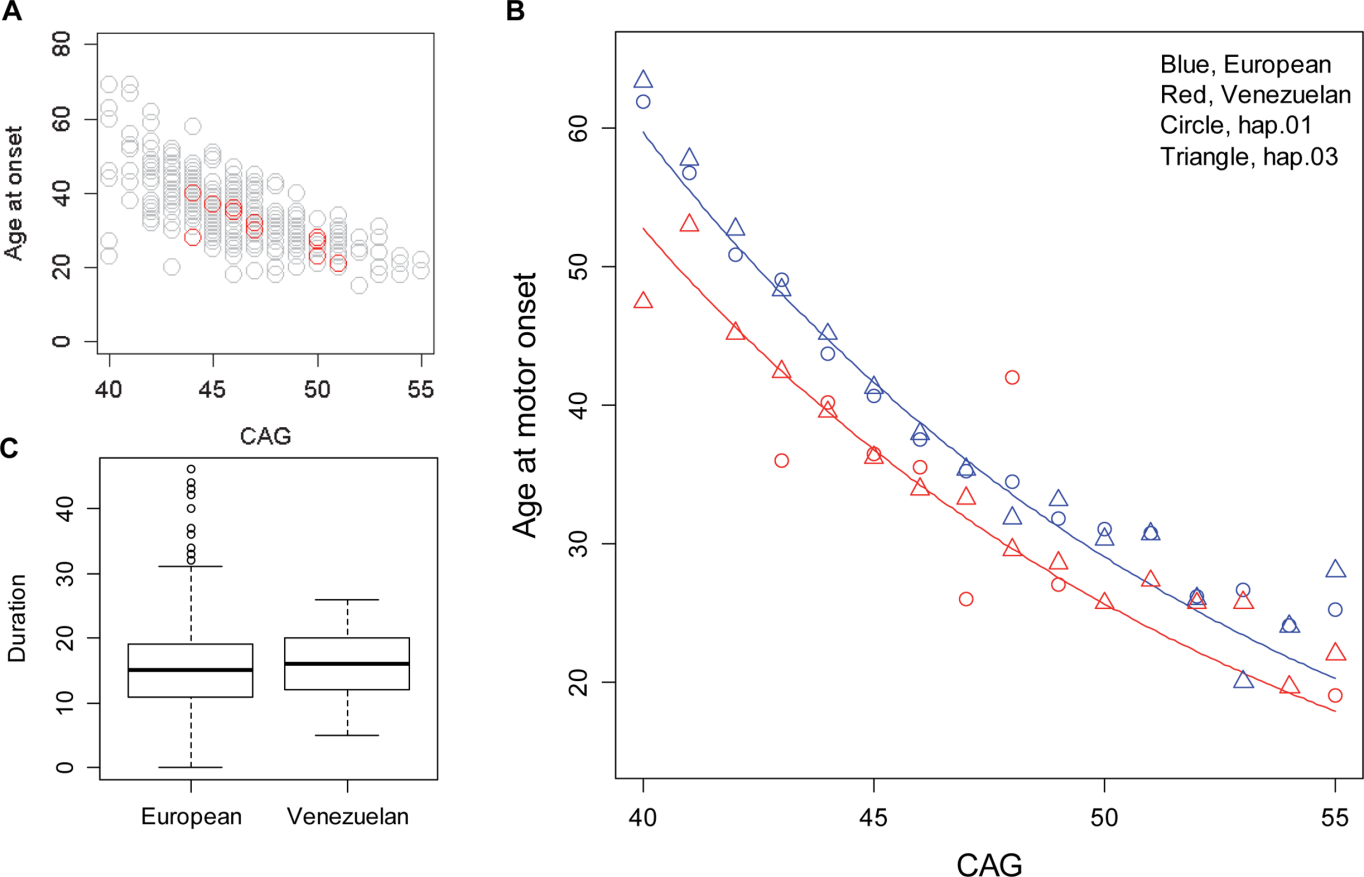
Earlier age at onset in HD subjects in Venezuela. (A) We evaluated the relationship between age at motor onset and the longer CAG repeat using the QC-passed data set. Age at onset and the longer CAG repeat of HD homozygotes are indicated in open red symbols (12 individuals). (B) Age at onset of motor signs was plotted against the size of expanded CAG repeat for HD subjects with European ancestry (blue) and Venezuelan HD subjects (red). Subsequently, log transformed age at onset (natural log) was modeled as a function of CAG repeat length for each population in a linear regression model. Then, fitted values were transformed back into natural scale age at onset for plotting. Blue and red lines represent regression models for European and Venezuelan HD, respectively. In addition, for a given CAG repeat size, the average of age at onset of HD subjects carrying a hap.01 disease haplotype (circle) or a hap.03 disease haplotype (triangle) are marked. (C) Disease duration data were compared between European and Venezuelan HD subjects. Duration values were available for 645 European HD and 85 Venezuelan HD subjects. Means of duration were 15.4 and 15.7 for European and Venezuelan HD subjects, respectively (Wilcoxon test p-value = 0.2783).

The mean CAG sizes across all haplotypes among the Venezuelans were 45.9 and 19 for longer and shorter alleles, respectively, and the average age at onset was 35.5 (S4 Fig). The expected decrease in onset age with increasing repeat length was readily observed in this data set (Fig 1A). Notably, 12 subjects carried two expanded alleles (Fig 1A; red circles), and, as predicted from our previous European-based analysis [7], their ages at onset were best explained by the longer allele in each individual (Fig 1A; shorter allele p-value in a regression analysis, 0.111), supporting full dominance. Interestingly, as noted previously [32], the age at onset of Venezuelan HD subjects was distinctly earlier than that in European HD subjects (Fig 1B; ~5 years earlier). While such a difference could theoretically occur due to systematic biases in calling motor onset in different countries/study populations, we believe this is unlikely because the Venezuelan HD subjects show a disease duration (the time interval between onset and death, which is CAG-independent) indistinguishable from European HD (Fig 1C; Wilcoxon test p-value = 0.2783) [21]. The consistency of mean disease duration across populations [21,42] argues that the earlier disease diagnosis for Venezuelan HD subjects does not represent a systematic calling of the diagnosis earlier in the disease process, which would result in longer durations, but rather truly reflects a more rapid CAG-dependent disease pathogenesis leading to motor onset.

Among European HD subjects, residual age at onset (i.e., observed age at diagnostic motor onset minus expected age at onset based on individual CAG repeat size) was not different between HD subjects carrying different common haplotypes [34], and the *HTT* locus did not emerge as associated with this phenotype in our GWA study of variations of >1% allele frequency [28]. For example, as summarized in Fig 1B, age at onset of European HD subjects with hap.01 (the major European disease haplotype, blue circles) is very similar to that of European HD subjects with hap.03 (blue triangles). Similarly, although based on a small number of samples, the most frequent Venezuelan haplotype hap.03 disease chromosomes (red triangles) did not appear to be consistently associated with earlier age at onset compared to Venezuelan hap.01 disease chromosomes (red circles). These data suggest that the striking difference in age at onset between European HD and Venezuelan HD subjects is not likely to be due to a *cis*-factor on the hap.03 *HTT* haplotype.

### Full sequence analysis of the Venezuelan hap.03 *HTT* haplotype

**Fig 2:**
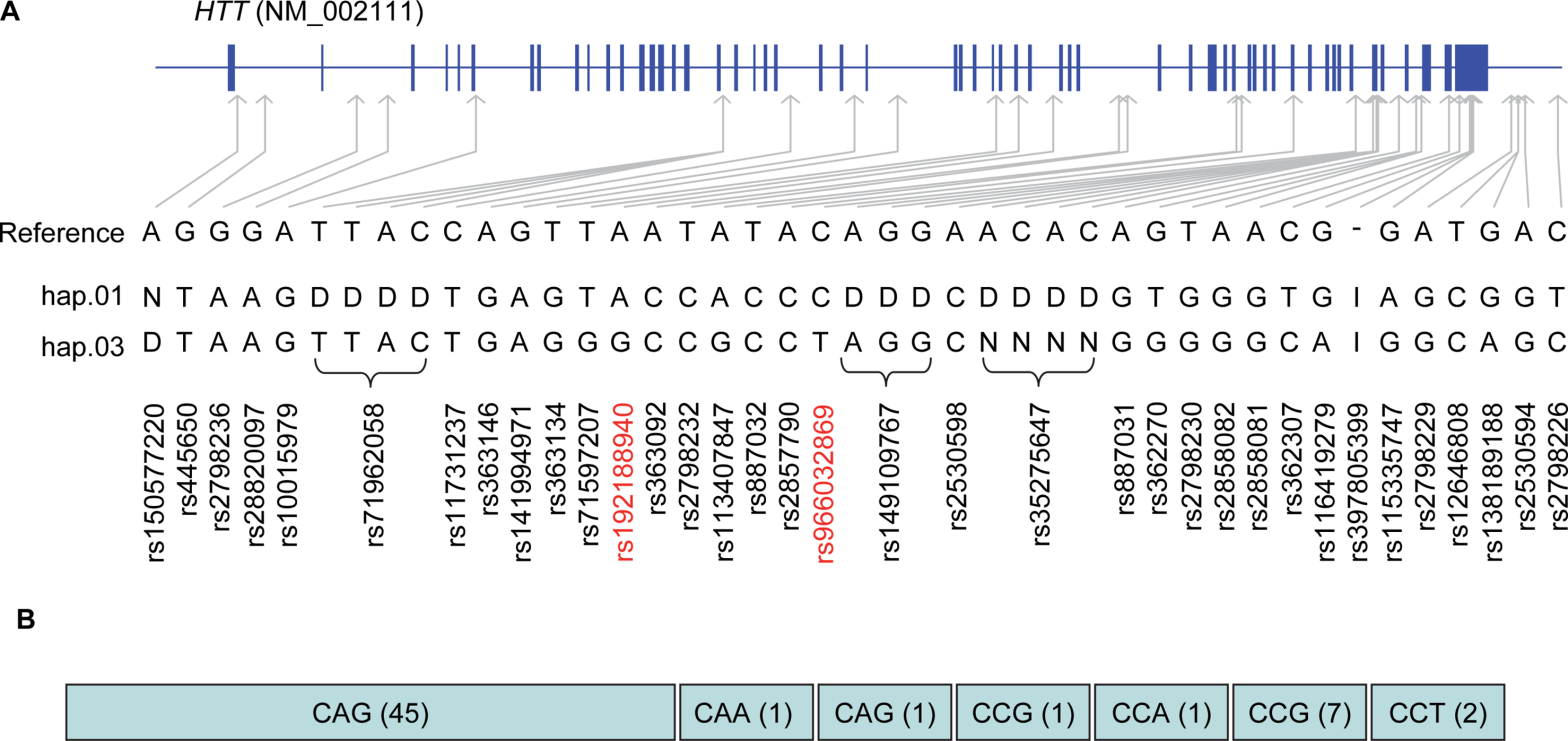
Sequence of hap.03 mutant haplotype. (A) Based on consensus alleles in 7 phased haplotypes in the sequencing family, the hap.03 disease haplotype sequence was reconstructed at the nucleotide level. Alleles on hap.03 that are different from the reference allele are shown with their genomic locations relative to *HTT* refseq NM_002111. The previously reported sequence of hap.01 is also displayed. D, I and N represent deletion, insertion, and missing, respectively. (B) The region containing the CAG repeat was missing in the sequencing data due to difficulty in aligning repeats. Thus, we performed additional Sanger sequencing to determine the sequence of the *HTT* CAG repeat region and found that the transmitting HD parent has a typical repeat sequence and downstream elements: uninterrupted 45 CAG repeats, 1 CAA, 1 CAG, 1 CCG, 1 CCA, 7 CCG, and 2 CCT, comparable to the structure seen on the most frequent HD hap.01 chromosome in Europeans.

To more directly assess the possibility that the HD-associated Venezuelan hap.03 chromosome carries novel genetic variations in *HTT* that are responsible for earlier onset than in Europeans, we determined the *HTT* genomic sequence by whole genome sequencing of a representative nuclear Venezuelan HD family from Family 1 (S1 and S5 Figs), carrying hap.03 as the mutant haplotype. This nuclear family comprises 2 parents and 7 offspring (S5 Fig), so independently phased sequences of each trio (S6 Fig) were merged to define the consensus allele of the mutant HD chromosome at each site (Fig 2A). Except for some repeat regions and a small number of inconsistent sites (S6 Fig), the sequence analysis determined 99.02% of *HTT* bases of the Venezuelan hap.03 HD disease chromosome (Fig 2).

Compared with the hg19 reference sequence (chr4:3066408–3255687; hg19 coordinates), the Venezuelan hap.03 disease chromosome carries alternative alleles at 28 variable sites, including a novel SNP (rs966032869 in intron 56) that was not described previously in public databases (Fig 2A). None of the variants at sites where Venezuelan hap.03 HD subjects carry non-reference alleles: 1) changes an amino acid, 2) is located in a splicing site, or 3) is associated with altered *HTT* mRNA expression levels (based on GTEx portal; https://www.gtexportal.org/home/). Additional Sanger sequencing analysis of *HTT* exon 1 revealed that the affected parent of this family (S5 Fig) carries a typical codon configuration in the polyglutamine/polyproline region consisting of 45 pure CAGs, 1 CAA, 1 CAG, and downstream elements of 1 CCG, 1 CCA, 7 CCGs, and 2 CCT codons (Fig 2B). These data collectively add to the argument that variations at the *HTT* locus are not likely to underlie the earlier age at onset in the Venezuelan HD subjects and suggest that this difference from European HD subjects may instead be due to unknown Venezuela-specific genetic and/or environmental factors [32].

### The origin of the CAG expansion mutation

Our full sequence data provide an unprecedented opportunity to assess the origin of the CAG expansion mutation currently shared by most subjects in the Venezuela HD cluster. Consequently, we compared the full *HTT* sequence of the Venezuelan hap.03 HD chromosome to European hap.03 disease chromosomes from HD subjects with Northern European, Spanish, and Portuguese ancestries (S7 Fig). The European hap.03 HD chromosomes do not differ from each other and are identical to the Venezuelan hap.03 HD chromosome at all but 2 sites: the novel SNP (rs966032869) and one annotated rare SNP (rs192188940) (S7 and S8 Figs). Interestingly, examination of these sites in a Brazilian HD subject carrying hap.03 revealed identity with the European hap.03, supporting an independent origin of the Venezuelan hap.03 HD chromosome.

Next, we compared the Venezuelan HD hap.03 sequence to 1000 Genomes Project data (Phase 3) to identify normal control subjects who may have identical sequences. Among various populations in the 1000 Genomes Project data, including Africans, Ad Mixed Americans, East Asians, Europeans, and South Asians, only one Mexican individual (NA19774) has sequence identical across 5864 comparable variation sites to the Venezuelan hap.03 disease chromosome, including at rs192188940 but excluding the novel SNP, rs966032869. The presence, but extreme rarity (MAF<0.001 based on UCSC Genome Browser) of rs192188940 on both normal chromosomes in the general population and on the Venezuelan hap.03 HD chromosome is most consistent with the CAG expansion mutation having occurred on a normal hap.03 chromosome that already carried the minor allele of this SNP. This normal chromosome may also have carried the rs966032869 minor allele or the latter may have arisen as a private mutation after CAG expansion. In any event, the most parsimonious explanation is that the major HD chromosome in the Venezuelan HD cluster arose by an ancestral CAG expansion event distinct from that associated with the common European hap.03 haplotype, potentially in an individual already geographically located in South America.

### Foundation for therapeutic strategies

We recently reported for the major European HD haplotype, hap.01, that an allele-specific CRISPR/Cas strategy based upon pairs of PAM site-altering SNPs could be used to inactivate the mutant *HTT* allele while leaving the normal *HTT* allele intact [43]. To provide the basis for future exploration of this potential therapeutic approach to benefit the Venezuelan HD population, we capitalized on the full sequence data to reveal variation sites suitable for mutant allele-specific silencing in this CRISPR/Cas strategy. Comparison to the haplotype most common in the normal population (i.e., hap.08 haplotype in 1000 Genomes Project, Phase 3 data) revealed Venezuelan hap.03-specific NGG CRISPR/Cas9 PAM-sites. For example, two CRISPR gRNAs depending on PAM-sites generated by rs2857935 and rs7659144 on the Venezuelan hap.03 would result in excision of the transcription start site and first two exons of the mutant gene, completely preventing generation of mutant *HTT* mRNA (S9 Fig; red arrows). This sequence-based precision medicine strategy holds promise for HD and other dominant disorders [43], and the definition of CRISPR/Cas PAM sites specific to hap.03 ensures that if substantial social, political, logistical and financial hurdles are overcome the development and consequent benefit of completely allele-specific gene targeting treatment strategies need not be limited on scientific grounds to European populations but could be extended to the Venezuelan HD cluster.

### Identification of genetic modifiers of disease onset in the Venezuelan HD population

**Fig 3:**
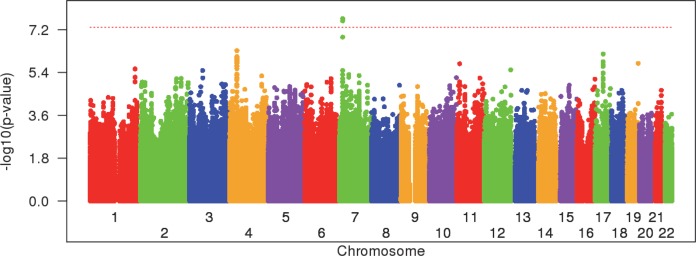
Genetic modifiers in Venezuelan HD subjects. Mixed effect linear regression analysis was performed for each SNP to determine the level of significance of the SNP in explaining the variance in residual age at onset in Venezuelans. SNPs that have minor allele frequency greater than 5% are shown, revealing genome-wide significant signals on chromosome 7. The X-axis and Y-axis represent chromosome and -log10(p-value), respectively. A dotted red horizontal line marks the level for genome-wide significance.

**Fig 4:**
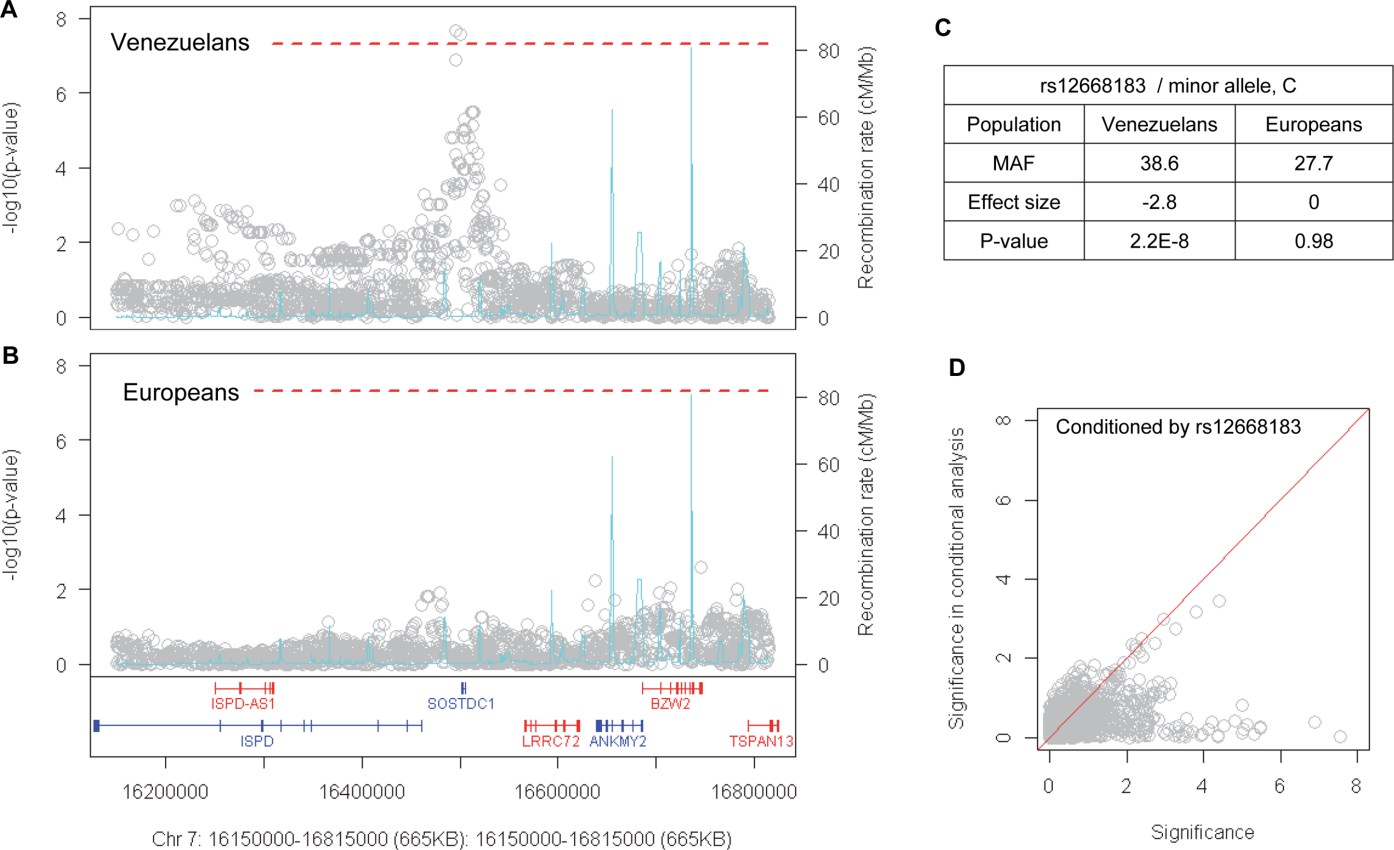
Genome-wide significant modification signals on chromosome 7 in Venezuelan HD. Association analysis results in Venezuelan HD (A) and in European HD (B) are displayed for the chromosome 7 region. Each grey circle represents a SNP. Y-axis and X-axis represent significance (-log10(p-value)) and genomic coordinate (hg19 assembly), respectively. Dotted red lines mark the level for genome-wide significance. Genes on the plus strand and negative strand are displayed in red and blue, respectively. Recombination rate based on HapMap data is shown as a blue trace (secondary Y-axis). (C) For the top SNP in this region in Venezuelan HD analysis, allele frequency, effect size, and p-values are shown for both Venezuelan HD and European HD. (D) In addition, a conditional analysis was performed focusing on the chromosome 7 region in Venezuelan HD subjects to determine whether a cluster of significant SNPs in this region tags a single modification signal. SNPs in this region were conditioned by the top SNP in the association analysis. Y-axis and X-axis represent significance in the conditional analysis and original association analysis.

Population stratification makes a genome-wide approach to identify genetic factors that are responsible for the earlier age at onset in combined Venezuelan and European HD subject cohorts problematic. However, the broad distribution of age at onset within the Venezuelan HD cohort does allow for statistical investigation of genetic modifiers present within this population using a GWA strategy. Consequently, we set out to identify genetic modifiers of HD in the Venezuela cluster by modeling residual age at diagnostic onset as a function of a SNP genotype and other covariates in linear mixed effect models to correct for familial relationships. Residual age at onset represents the difference between observed age at onset and expected age at onset predicted from CAG repeat size in this population (Fig 1B, red line), thus reflecting the degree of modification of onset age by factors other than CAG repeat size (S10 Fig). In a GWA analysis of 374 Venezuelan HD individuals (Fig 3; S11 Fig), we observed one genome-wide significant modifier locus on chromosome 7 (Fig 4) and loci of suggestive significance on chromosomes 4 (S12A Fig) and 17 (S12B Fig). Considering the small sample size in our genetic analysis, detection of genome-wide significant signals was surprising, although common modifiers may show stronger effect sizes than typical common disease risk alleles in GWA studies [28]. In any event, additional analyses argue against statistical inflation (S11 Fig).

### Population-specific modification signals

Interestingly, the chromosome 7 locus that shows genome-wide significant association signals in the Venezuelan HD subjects (Fig 4A) was not significantly associated with residual age at onset in European HD subjects (Fig 4B). The same is true for suggestive significant loci on chromosomes 4 and 17. The top SNP at the chromosome 7 locus, rs12668183, is associated with a -2.8 year effect size per minor allele in Venezuelan HD, but no effect in European HD subjects (Fig 4C; S13 Fig), indicating population-specific modification of HD. Intrigued by this, we performed genetic modification score analysis using a polygenic risk score method to compare these HD populations. Based upon association analysis results from European HD subjects [28], we constructed a polygenic modification scoring routine using effect sizes of 44 independent suggestive SNPs (p-value < 0.00001) and the number of effect alleles of scoring SNPs. Then the same scoring method was applied to the training set (European HD subjects) and the Venezuela test set to judge how well the European-based HD modification score explains phenotypic variance in the Venezuelan HD cluster. As anticipated, the European polygenic modifier score explained a significant amount of variation in residual age at onset among European HD subjects (S14A Fig; modifier score p-value < 2E-16; R-squared, 19.9%). However, the modification score did not explain the variance in residual age at onset in the Venezuelan HD subjects (S14B Fig; modifier score p-value, 0.75; R-squared, 0.03%). Consistent with the chromosome 7 modification signals, this difference may reflect a major contribution of population-specific factors to modification of HD.

**Fig 5:**
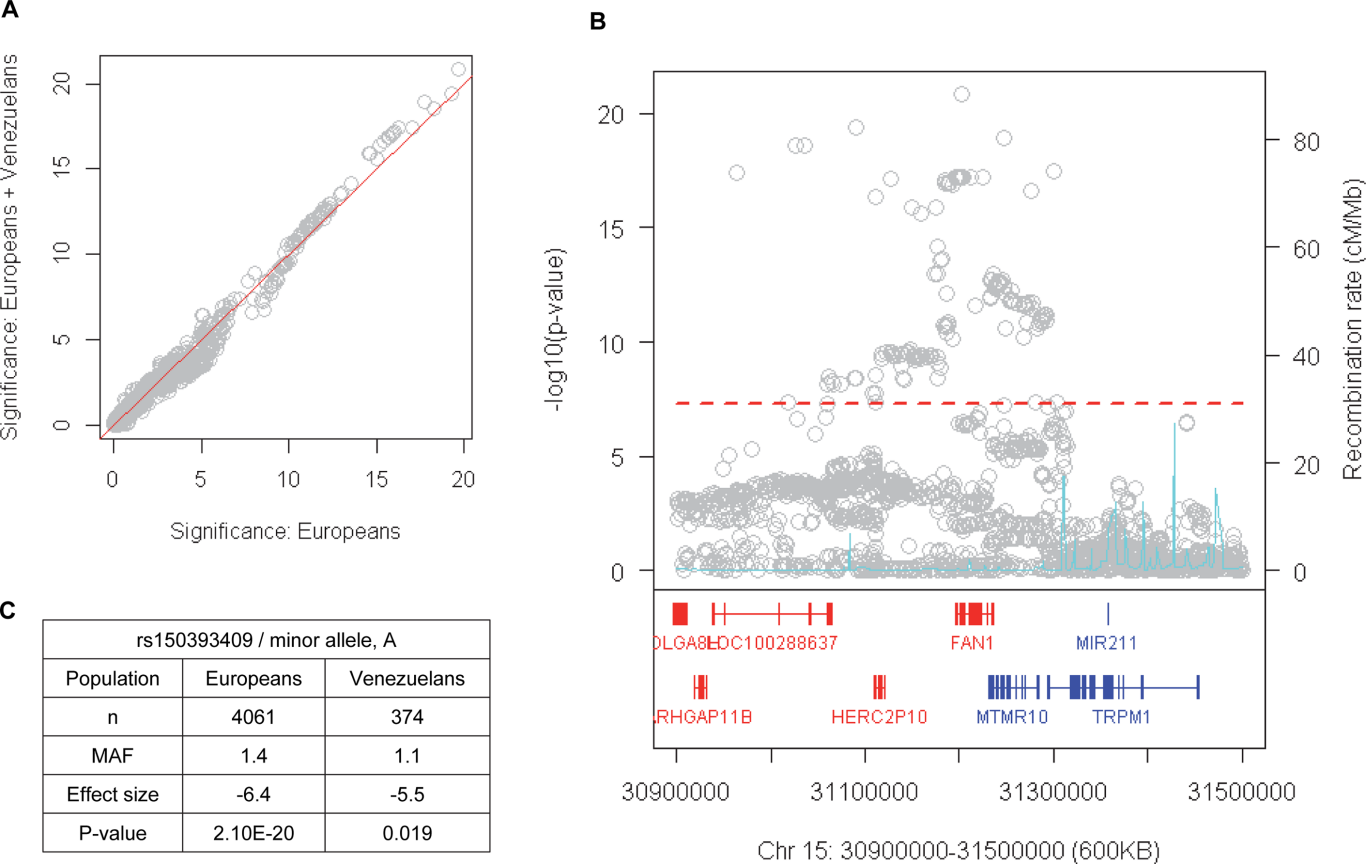
Improvement of chromosome 15 association signals by addition of Venezuelan HD data. (A) GWA analysis results for European HD subjects were combined with those of Venezuelans in order to determine whether or not addition of Venezuelan data improved modification association signals. Association signals in Europeans (X-axis) and that in Europeans + Venezuelans (Y-axis) are plotted. Open grey circles, which are above the red diagonal line, represent SNPs whose significance in association analysis was improved when European and Venezuelan data were combined. (B) A regional association plot was generated focusing on chromosome 15, revealing that rs150393409, which specifies an arginine to histidine missense alteration predicted to be deleterious to FAN1, is the most significant SNP in this region. (C) A summary table is shown focusing on the top SNP in European + Venezuelan HD analysis.

The genetic modifier signals previously discovered on chromosomes 15, 8 and 3 in the larger European HD GWA study [28] were not detected in the Venezuela HD GWA sample (Fig 3). This is likely due to the reduced power of the much smaller Venezuela HD sample. Indeed, the association signals at those loci that were genome-wide significant in European HD subjects (both rare and common modifier signals) were modestly improved by adding Venezuelan HD subject data into the meta-analysis (Fig 5A), consistent with these known HD modifiers also acting to modify HD in Venezuelans. For example, on chromosome 15, when meta-analysis was performed to combine European HD data and Venezuelan HD data, the overall association signals were improved, revealing the strongest modification signal at the SNP rs150393409, a predicted deleterious missense alteration in *FAN1* that is likely to be the functional variant responsible for modification (Fig 5B). Effect sizes for this SNP were similar between the two populations (-6.4 and -5.5 years/minor allele, respectively) (Fig 5C). Together, our association analysis results indicate that there may be both population-shared and population-specific modifying genetic factors that influence the rate of pathogenesis in HD.

### Venezuelan-specific modification signals on chromosome 7

**Fig 6:**
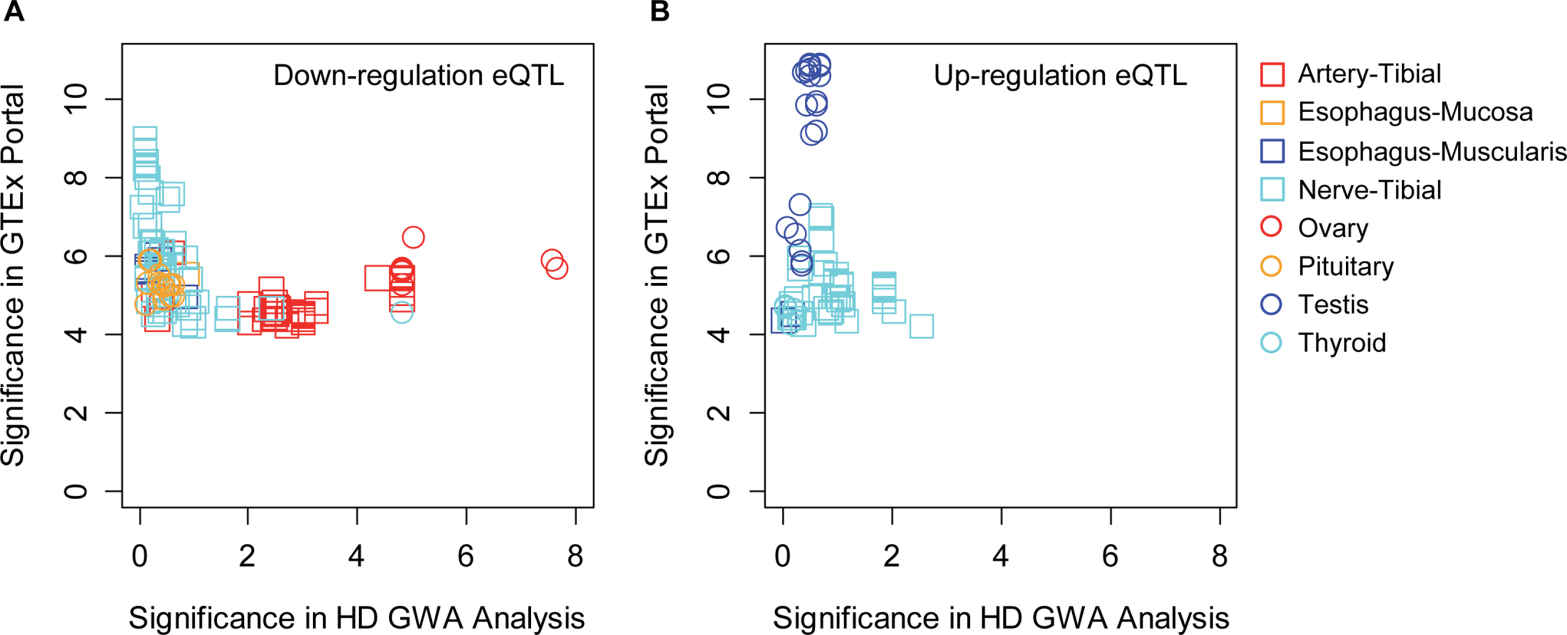
A potential role for reduced *SOSTDC1* expression levels in HD onset modification. Modifier-associated SNPs in the chromosome 7 region were checked for association with expression levels of *SOSTDC1*. The Y- and X-axes represent significances in GTEx eQTL and modification in our GWA analysis, respectively. Panel A shows SNPs whose minor alleles are associated with decreased expression levels of *SOSTDC1*. Panel B shows SNPS whose minor alleles are associated with increased expression levels of *SOSTDC1*.

Focusing on the Venezuelan-specific modification signals on chromosome 7, the top SNP in this region, rs12668183, is common in all 1000 Genome Project populations with minor allele frequencies ranging from 30% in Europeans and 35% in the Ad Mixed Americans to 46% in East Asians. When SNPs in this region were conditioned by rs12668183, no SNPs remained significantly associated (Fig 4D), indicating that the significant SNPs all tag a single modifier effect. The association signals were not contributed by CNV since the frequency of CNV in the region was very low (S15A Fig) and when samples carrying CNV were excluded from the analysis, association signals were virtually unchanged (S15B Fig). The association signals were not solely contributed by Family 1, since association analysis based separately on this family and on all other families as a group generated similar significance levels (S16 Fig), suggesting roughly equal contributions to the overall association signals. The association signals also were not due to skewing of the quantitative analysis by a few inaccurately phenotyped individuals since dichotomous analysis comparing allele frequencies of extreme residual age at onset groups was consistent with the continuous QTL GWA analysis result (S17 Fig). The associated SNPs tagged a single modifier haplotype at *SOSTDC1* (Fig 4), which encodes a member of the sclerostin family (https://www.ncbi.nlm.nih.gov/gene/25928) that functions as a bone morphogenetic protein (BMP) antagonist, and plays a role in signaling during cellular proliferation, differentiation, and programmed cell death [44–47]. Each minor allele of the top SNP was associated with a hastening of onset by 2.8 years compared to those homozygous for the major allele. The most significant SNPs associated with HD age at onset are located just 3’ to *SOSTDC1*, and also show significant eQTL (expression quantitative trait loci) signals for *SOSTDC1* in the GTEx data set (http://www.gtexportal.org/home/eqtls/byGene?geneId=sostdc1&tissueName=All) (Fig 6) whereas none of these SNPs show evidence of association with expression of any of the other genes in the region. Interestingly, the minor alleles of SNPs most significantly associated with modification of HD onset are associated with reduced expression of *SOSTDC1* in some GTEx tissues (e.g., ovary, artery-tibial, thyroid) (Fig 6A) but not with increased expression (Fig 6B). This relationship supports the hypothesis that the accelerated onset of HD caused by this modifier locus is due to reduced levels of *SOSTDC1*.

## Discussion

Investigation of haplotypes in HD subjects, including family members from the large Venezuelan HD cluster and many families from North America and Europe, originally permitted delineation of the chromosomal localization of the HD mutation [4–6,35], and ultimately led to identification of the cause of the disease [3]. The most frequent *HTT* haplotype on HD chromosomes, currently named hap.01 [34,38,48] and defined by a standard panel of frequent SNPs, accounts for the largest proportion of HD subjects of European ancestry [34,37,38], but CAG expansions also occur on a variety of other haplotypes [33–38], indicating that multiple mutational events have produced HD and emphasizing the unstable nature of *HTT* CAG repeats [16,24,49,50]. The third most frequent *HTT* disease haplotype among HD subjects throughout Europe is hap.03. Consistent with our observation of a Brazilian subject with a hap.03 HD chromosome comparable to that in Northern European, Spanish and Portuguese subjects, *HTT* CAG expansion mutations of HD subjects in Latin America have been hypothesized to be of European origin [51]. However, sequence differences on the Venezuelan HD cluster hap.03 disease chromosome are most consistent with an independent origin of their HD mutation, possibly due to CAG expansion in an individual in South America. With the full sequence of the Venezuelan HD chromosome now available, a finer definition of the origin of this disease mutation may ensue from the accumulation of more extensive genomic data from geographically defined normal populations. Similar studies of other geographically separated HD clusters could also provide further insights into how normal range CAG repeats expand into the disease-causing range, the emergence of new cases of HD and genetic anticipation.

The discovery of the cause of HD made possible the generation of model systems [22] and numerous hypotheses regarding disease mechanisms have subsequently been raised [1,9]. However, many mechanism-based therapeutic targets that were highly efficient in animal models have turned out not to be effective in humans [52,53], perhaps due to species-specific differences in the effects of, or in the response to, the HD mutation or to the use of extreme CAG repeat lengths in most models. An alternative route to developing rational treatments is to capitalize on the genetic information that can be obtained from studies of human HD subjects. We have pursued two distinct directions in this regard, both of which are augmented by our findings concerning the Venezuelan HD cluster. The most readily definable therapeutic target in HD is the mutant gene itself, since elimination of its expression would remove the factor that ultimately precipitates all aspects of the disease. Our previous sequence analysis of hap.01, the major European haplotype, enabled us to develop allele-specific CRISPR/Cas targeting strategies [38,43,48] to inactivate the mutant *HTT* allele without damaging the normal *HTT* allele, providing a novel route to developing a treatment. The full sequence of the Venezuelan hap.03 HD chromosome and consequent definition of mutant allele-specific CRISPR/Cas9 PAM sites supports this same approach, ensuring that further therapeutic development of these allele-specific inactivation strategies need not be limited on scientific grounds to European HD subjects, but, if societal hurdles were overcome, could also benefit those in the Venezuelan HD cluster who have contributed so much to HD research. We have also reasoned that more traditional pharmaceutical development for HD could benefit from rational, in-human validated, therapeutic targets discovered by identifying genes that act to significantly modify disease pathogenesis in the subjects themselves. The well-established inverse relationship between CAG repeat length and age at clinical onset of HD pointed both to the CAG repeat size as the driver of the rate of HD pathogenesis and to a role for genetic and environmental factors in modifying the impact of the CAG repeat [7,15–18,30–32,54]. Our haplotype analyses indicated that other genetic variations at *HTT* are not a common source of HD modification [34], yet the variance in age at onset at any given repeat length is large and heritable, so we pursued a GWA analysis of subjects of European ancestry to discover significant unlinked modifiers of the age at clinical onset. The most significant locus hastens onset by approximately 6 years per rare allele demonstrating that quite strong modifier effects are possible in humans [28]. These modifier effects are due to naturally occurring variations in particular genes and pathways, and it can be expected that pharmaceuticals developed to target the same pathways could have even larger effects.

Our initial European HD GWA analysis and follow-up study highlighted the importance of DNA maintenance/repair pathways in modifying the rate of CAG-dependent HD pathogenesis and suggested that somatic instability of the CAG repeat accelerates the disease [28,40]. While we saw some evidence that these processes also act to influence HD onset in Venezuela, a much stronger result from our unbiased genetic study of Venezuelan HD subjects points to a different modifier process. The genome-wide significant modification signals are focused at *SOSTDC1* (sclerostin domain containing 1) whose protein product functions as a bone morphogenetic protein (BMP) antagonist [46,55] and therefore negatively regulates BMP signaling. The expression of this gene is altered in various cancers [45,56–58]. The locus is also associated with lipid-lowering in response to statins [59] (https://www.ebi.ac.uk/gwas/), providing a clear example of a phenotype determined by gene-environment interaction.

Compared to European HD, the Venezuelan HD subjects display a similar CAG size/age at onset relationship, and full dominance of the longest CAG allele, but at equivalent CAG sizes, they develop motor symptoms earlier, suggesting the potential for population-specific genetic or environmental influences that accelerate HD pathogenesis. Our GWA analysis appears to have detected one such locus, whose combination of high allele frequency (38%) and large effect size (almost 3 years earlier onset per minor allele) made it detectable at genome-wide significance in only a few hundred Venezuela subjects. Population-specific modifiers of a disease have been reported only rarely in the literature [60] but examples of population-specific disease risk association are reported frequently [61–67]. Population-specific studies can therefore provide deeper insights into genetic architecture of human traits and diseases, although the relative contribution of population-specific variants, gene-gene interactions and gene-environment interactions has not been fully assessed. The fact that the chromosome 7 HD modifier locus does not yield evidence of modification in European HD suggests that the modifier effect is due either to a Venezuela-specific functional variant at the locus or to interaction of the locus with some genetic or environmental feature specific to the Venezuelans. Assessment of the former possibility will require detailed sequence comparison and functional dissection of the locus, but the observation that the top SNPs are also associated with reduced expression of *SOSTDC1* in non-Venezuela data suggests that the second alternative is also worthy of consideration. Our data are consistent with the possibility that reduced expression of *SOSTDC1* in individuals carrying the chromosome 7 modifier haplotype interacts with a population-specific factor to modify HD in Venezuela. A role for shared environmental factors in influencing age at onset in the Venezuelan HD cluster has been proposed previously [32] and in an initial assessment of potential genetic interaction involving the chromosome 7 locus, we detected suggestive signals (S18 Fig) that might reflect functional genetic interaction. Identification of the Venezuela-specific factor at the chromosome 7 locus, at an interacting locus, or in the environment that contributes to the modifier effect could yield insight into the mechanism of HD and provide another route to treatment that might be applicable in both the Venezuelan and other HD populations. Interestingly, SNPs whose alternative alleles are associated with increased levels of *SOSTDC1* have much lower allele frequencies among our study subjects, potentially explaining the failure to yield significant signals in the modifier GWA. However, those alleles do show trends toward positive modification, implying that increased levels of *SOSTDC1* may delay age at onset in this population. From this perspective, identification of factors that induce increased levels of *SOSTDC1* may also have therapeutic utility.

In summary, our comprehensive genetic investigation of a distinct disease population has revealed intriguing aspects of HD, which are directly relevant for gene-based and traditional target-directed small molecule therapeutic development, both for this population and potentially for HD more generally.

## Methods

### Ethics statement

The study was approved by the Partners HealthCare Institutional Review Board as Protocol #: 2009P000566/PHS, “Genetic Variation that Modifies HD Phenotype” and performed on DNA samples deposited previously, based upon oral consent, into a Partners HealthCare Institutional Review Board approved repository (currently Protocol# 2010P001611/PHS, “CHGR Neurodegenerative Repository”).

### Subjects, genotyping, quality control analysis, and genotype imputation

In a study approved by the Partners HealthCare Institutional Review Board, the Genetic Modifiers of HD (GeM-HD) Consortium carried out genotyping for 3,447 HD subject samples from the Massachusetts Huntington’s Disease Center without Walls repository, which included banked samples from North American, European and Venezuelan families who had previously donated blood samples for HD research, and from the European Huntington's Disease Network (EHDN) Registry study [68]. Data from Illumina Omni2.5 arrays were generated at the Broad Institute and European HD subjects from this dataset (namely, HD GWA3) were analyzed with previous HD GWA data in order to identify genetic modifiers of HD in Europeans [28]. 549 subjects from the Venezuela HD cluster, described in Wexler *et al*., 2004 [30], were excluded from the previous analysis due to ancestry, and these form the primary population investigated in this study. As previously described [28], we applied quality control (QC) metrics such as SNP call rate >95%, minor allele frequency (MAF) >1%, Hardy-Weinberg equilibrium p-value >1E-6, sample call rate >95% to prepare high quality genotype data (538 subjects). Ancestry of samples was confirmed by principal components analysis using the PLINK program [69]; MDS (multidimensional scaling) values of study subjects were compared to those of 1000 Genome Project samples. Familial relationships were determined based on pair-wise IBD (identity-by-descent) estimation using the PLINK program and compared with existing pedigree charts from the U.S.-Venezuela Collaborative Research Project [32]. For a given individual, all related individuals with PLINK PI_HAT value greater than 0.125 were identified, and grouped into a family. Subsequently, we checked if the rest of samples were related to any of members of that family based on the same PI_HAT value criteria, and newly discovered related individuals were merged with previously defined family. These procedures were repeated exhaustively to identify all related subjects, generating 22 families with variable sizes. Therefore, an individual within a given re-constructed family is related to at least one other subject. Since we used estimated IBD values in identifying relatives to re-construct families, some families may have somewhat distantly related members. Haplotype phase of study subjects was based on computational phasing of 21 tagging SNPs [34,38], generating the names of haplotypes for each individual (e.g., hap.01 - hap.16). Since all HD subjects in each family share the same ancestral HD disease haplotype, the most frequent haplotype in the family was assigned as the HD disease chromosome haplotype. Genotype imputation was performed by the Michigan Imputation Server (https://imputationserver.sph.umich.edu/index.html) using the 1000 Genomes Project mixed population as a reference panel. The SHAPEIT phasing program (https://mathgen.stats.ox.ac.uk/genetics_software/shapeit/shapeit.html) was used for pre-phasing study samples. The same QC parameters were applied to imputed data to ensure the quality of genotype data.

### CAG repeat size, age at onset of motor symptoms of HD, and residual age at onset phenotype for genetic analysis

Methods to determine the sizes of *HTT* CAG repeats for each Venezuelan study subject are described elsewhere [70]. Age at onset was determined either prospectively or retrospectively based upon examination of subjects by experienced neurologists as described by the U.S.–Venezuela Collaborative Research Project [32]. Regression modeling analysis was performed to describe the specific relationship between age at onset and CAG repeat size in these Venezuelans. Briefly, natural log transformed age at onset of Venezuelan HD and European HD were modeled as a function of the size of expanded CAG repeat; study population-specific intercepts were estimated by including a covariate specifying individual population in the regression model. When a HD subject carries two expanded alleles (CAG > 35), the longer one was used because of complete dominance [7]. This regression model described the overall relationship between CAG and age at onset, and still permitted the revelation of any differences between Venezuelan HD and European HD. Using this regression model, predicted age at onset was estimated based on CAG repeat size, and subsequently was subtracted from actual age at onset to calculate residual age at onset for each HD individual. Residual age at onset represents how early or late a HD subject develops motor symptoms compared to expectation based on his or her expanded CAG repeat length. A positive residual age at onset means an HD individual developed symptoms later than expectation, and a negative residual age at onset means an HD individual developed symptoms earlier than expectation.

### Whole genome sequencing and phasing

For a selected nuclear family, where the HD mutation was transmitted on the hap.03 haplotype, we performed whole genome sequencing analysis as described previously [48]. Sequencing was performed by Complete Genomics, generating variant calling files containing sequence variants with confidence scores. Since we aimed at maximizing SNP discovery and the inclusion of related samples provided the opportunity to assess sequencing errors, we examined all variants originally reported by Complete Genomics. For this study, we focused on a genomic region (chr4:3066408–3255687; hg19 coordinates). Haplotype phasing was performed by using father-mother-child trio data as summarized in S5 Fig. Seven trios with a HD parent, normal spouse and HD child were defined in this family, and therefore the same parents were members of 7 trios, but data pre-processing and trio phasing was performed for each trio independently. Briefly, a variant site was excluded in a given trio if 1) genotypes in all 3 individuals were unknown, 2) all 3 individuals were identically heterozygous or 3) a Mendelian error was detected. Since we aimed at maximizing variation coverage, we included sites with partially missing data even though this makes detection of Mendelian errors difficult. Thus, Mendelian error detection was based on checking the genotype data for the following: 1) variant sites without any missing data, 2) sites with no missing data in the child and one or two alleles missing in only one parent, and 3) sites with one allele missing in the child and none missing in parents. This detection pipeline could still miss certain Mendelian errors due to missing genotypes, but these errors could be further identified by subsequent merging of multiple phased haplotypes in the family. After removing sites with Mendelian errors, sites that could not be confidently phased due to missing genotypes, and sites heterozygous in all 3 members of the trio, we phased the alleles at all remaining sites to produce fully phased hap.03 disease haplotype for each trio.

After data pre-processing, trio genotype data were phased using the BEAGLE program (https://faculty.washington.edu/browning/beagle/beagle.html), and the HD disease chromosome haplotypes were further analyzed. One hap.03 disease chromosome haplotype, inherited from the father, was obtained for each of the seven trios, and we merged these to discover any inconsistent alleles for additional QC analysis. Locations and numbers of Mendelian errors and inconsistent alleles are summarized in S6 Fig. We then finalized the hap.03 disease haplotype by 1) taking alleles for sites with at least two phased allele calls, 2) assigning ‘N’ for sites that were unphaseable, or had missing genotype or Mendelian errors, and 3) assigning ‘?’ for the inconsistent sites. These procedures yielded phased haplotypes covering 99.02% of the sites in the region. A novel SNP variation was identified, and submitted to dbSNP Build 150 (rs966032869).

### Capture sequencing analysis of Venezuelan and European HD subjects

In order to validate Venezuelan HD whole genome sequencing data and compare to the hap.03 disease chromosome in European HD subjects, we performed capture sequencing for an additional 8 Venezuelan HD subjects and 7 unrelated European HD subjects. Haplotypes of disease and normal chromosomes were based on computational phasing [34] of our GWA samples [28]. Capture probes were designed to enrich the target region chr4:3066408–3255687 (hg19 coordinates) using the Agilent SureDesign online tool. This generated ultra-long 120-mer biotinylated complementary RNA baits, and these capture probes were used for solution-based SureSelect target enrichment. Briefly, genomic DNAs were sheared to produce smaller fragments and libraries prepared with sequencer specific adaptors and indexes for multiplexing. DNA libraries were hybridized with biotinylated cRNA baits, which were complementary to regions of interest. The bait-library complexes were pulled down by magnetic beads. After the beads were washed, the RNA bait was digested to obtain the target DNA of interest, and subjected to sequencing using Illumina HiSeq 100bp paired-end sequencing at the Broad Institute. Sequence reads were aligned for variant calling using the Genome Analysis Toolkit Best Practices workflow (https://software.broadinstitute.org/gatk/). Additional genotyping for specific sites was performed by conventional Sanger sequencing.

### Genome-wide association analysis, conditional analysis, and extreme dichotomous analysis

Residual age at onset was modeled by SNP, gender, and ancestry covariates (i.e., MDS 1–4) in a linear mixed effect model based on kinship matrix using the GEMMA program (http://www.xzlab.org/software.html). We applied an additive model for each SNP whose minor allele frequency is greater than 5% in the study population. GWA analysis using imputed genotypes revealed genome-wide significant SNPs at a locus on chromosome 7. Subsequently, we compared genotypes (chr7: 16150000–16815000, hg 19) of 5 Venezuelan HD individuals who had both sequence and imputed genotype data, revealing a mean concordance rate of 98.7% (range, 98.2 ~ 99.3%).

Genomic inflation factor was calculated using the GenABEL package (median method) (http://www.genabel.org/manuals/GenABEL). Recombination rate was obtained from HapMap data (http://www.sanger.ac.uk/resources/downloads/human/hapmap3.html), and plotted on the secondary Y-axis of regional association plots. In order to determine dependency of SNPs in a given region, linear mixed effect models were constructed similarly but included the most significant SNP as a covariate. Significances of SNPs in the ordinary association analysis were compared to those in conditional analysis. Also, we performed extreme dichotomous analysis to determine whether a small number of data points drove the significant association. We directly compared SNP allele frequencies of those individuals whose residual age at motor onset was among the 10% extremes of earlier and later than expected onset. Logistic regression analysis was performed using the GEMMA program.

### CNV analysis, meta-analysis, and polygenic modification score analysis

Genome-wide copy number variation was determined from GWA data using PENNCNV program (http://penncnv.openbioinformatics.org/en/latest/). For chromosome 15 region, we meta-analyzed association analysis results of Venezuelan HD and European HD using the METAL program (https://genome.sph.umich.edu/wiki/METAL_Documentation). In order to determine whether a polygenic modification score based on European HD subjects captured individual deviation from the expected age at onset for a given CAG size in Venezuelan HD, we obtained effect sizes and significances of SNPs based on the European HD subjects. Polygenic modification score is defined as the sum of effect allele count X effect size for each HD subject. We used independent SNPs with suggestive significance (p-value < 0.00001). This scoring method was applied to European HD and Venezuelan HD to calculate individual modification score. Subsequently, polygenic modification score was used as the independent variable with gender and ancestry covariates to explain residual age at onset in each population.

### GTEx eQTL analysis

For SNPs in the chromosome 7 region, we evaluated the significance of eQTL with genes in the region using the GTEx portal data base (http://www.gtexportal.org/home/). Among *ISPD*, *SOSTDC1*, and *LRRC72*, *ISPD* and *SOSTDC1* generated significant eQTL signals in GTEx data set, and therefore these SNPs were downloaded for subsequent analysis. 42 and 231 SNPs (non-unique) were significantly associated with expression of *ISPD* and *SOSTDC1* in various human tissues, respectively. For those significant eQTL SNPs, we compared the significance (-log10(p-value)) in our modifier association analysis to those in GTEx *cis* eQTL analysis, revealing a correspondence between GWA significance and eQTL significance only for *SOSTDC1*.

### Potential genetic interaction with chromosome 7 modifier locus

Venezuelan HD subjects were separated into two groups based on chromosome 7 modifier genotype (rs12668183), resulting in rs12668183-carriers and non-rs12668183-carriers. For each group of Venezuelan HD subjects, independent GWA analysis was performed. In order to reduce outlier effects in the continuous analysis, subjects were sorted based on residual age at onset, and top and bottom 40% of subjects were analyzed using dichotomized phenotype data, focusing on comparing allele frequency differences between early and late onset groups. Mixed effect models were used to correct familial relationship.

## Supporting information

S1 Fig Ancestry characteristics of Venezuelan HD subjects. In order to confirm ancestry of Venezuelan HD study subjects, principal components analysis using genome-wide SNP data of samples was performed together with 1000 Genomes Project data (Phase 3; 2,504 samples from 26 populations). The first vs. second MDS (multidimensional scaling) values (top row), and the third vs. fourth MDS values (bottom row) were plotted. 1000 Genomes Project samples are represented by colored symbols (A and B), or open grey symbols (C, D, E, and F). Study subjects used for GWA analysis (C and D) or sequencing analysis (E and F) are indicated by open black squares. For detailed description of populations and symbols of 1000 Genomes Project, refer to http://www.internationalgenome.org/faq/which-populations-are-part-your-study/.
(PDF)

S2 Fig Familial relationships of study subjects. For QC-passed 374 Venezuelan HD study subjects analyzed in GWA analysis, familial relationships were inferred based on genome-wide genotype data. Pair-wise IBD (identity-by-descent) values were estimated by the PLINK program. From 374 unique study subjects, IBD values were estimated for 69,751 meaningful pairs. The mean of PI_HAT value (indicative of IBD) was 0.0253 (A). Based on Z1 and Z2 values from the PLINK IBD estimation, we identified 170 unique parent-child pairs comprising 216 subjects (B, red rectangle). In addition, siblings and other relationships were also revealed (B).
(PDF)

S3 Fig Reconstruction of families based on genome-wide genotype data. Based on pair-wise IBD estimations, families were reconstructed. A pair of related individuals whose PLINK PI_HAT value was greater than 0.125 (representing first cousin or closer relationships) were combined, and this process was repeated exhaustively until all related individuals were combined, generating 22 families with variable sizes. Therefore, an individual within a given re-constructed family has at least one relative. Families were numbered arbitrarily and were separated by blue boundary line. The size of each family is provided in a parenthesis, and haplotype of HD disease chromosome is indicated in parenthesis after slash. Unrelated individuals (18) are at the bottom-right of the plot without family name.
(PDF)

S4 Fig Descriptive statistics of Venezuelan HD study subjects. Following sample QC analysis, 374 Venezuelan HD subjects including 12 homozygote HD individuals were analyzed. Histogram plots show distributions of longer CAG repeats (A), shorter CAG repeats (B), and age at onset of motor symptoms (C). The means of longer CAG, shorter CAG, and age at onset of motor symptoms are 45.9 (A), 19 (B), and 35.5 (C), respectively.
(PDF)

S5 Fig HD nuclear family used for whole genome sequencing analysis. Nine subjects including one non-HD parent were used for whole genome sequencing. This family inherits the hap.03 haplotype on the mutant chromosome as the majority of Venezuelan HD subjects do. For each subject, haplotypes of mutant chromosome (red) and normal chromosome (black) are indicated. In addition, to determine the phased sequence of *HTT* in this family, we performed trio phasing for each trio comprising parents and a child. As indicated by "Trio #", 7 trios were used for phasing analysis. For each trio phasing a small number of Mendelian errors were detected. Diamond symbols were used for children to maintain confidentiality.
(PDF)

S6 Fig Summary of sequencing and phasing analysis of *HTT* locus. (A) For each trio, trivial phasing analysis was performed using the BEAGLE program, and 7 phased mutant chromosomes were merged to identify consensus alleles of hap.03 disease haplotype in this family. We detected 6 unique Mendelian error sites, 3,231 unphaseable sites due to missing data, and 7 inconsistent sites. In total 99.02% of bases were determined. Examples of inconsistent sites are shown in panel B. N and D represent missing and deletion.
(PDF)

S7 Fig Venezuelan mutant hap.03-specific sequence variations. (A) Northern (filled blue circles) or Southern European HD subjects (filled purple and orange circles representing Spanish and Portuguese HD, respectively) carrying hap.03 as the mutant chromosome were identified using phased haplotypes of GWA data. A scatter plot shows MDS 1 and 2 values from principal components analysis of GWA data relative to control populations such as CEU and TSI.
(B) Consensus alleles of the hap.03 haplotype in Venezuelan HD were obtained from trio phasing of family samples. In addition, consensus alleles of the hap.03 disease haplotype in 7 independent Northern European HD subjects were obtained from capture sequencing analysis (7 samples). Direct comparisons of consensus alleles revealed 2 sites (including a novel SNP) where alleles are different between Venezuelan and European hap.03 disease haplotypes. For the known SNP site (rs192188940), imputed GWA data for Spanish (15 samples) and Portuguese HD (43 samples) with hap.03 haplotype are also indicated. Since the novel SNP is not part of the 1000 Genomes Project reference panel, rs966032869 was not imputed in GWA data.
(PDF)

S8 Fig Validation of Venezuelan HD-specific variation sites. Sanger sequencing analysis was performed to validate the genotypes of Venezuelan hap.03-specific SNPs including a novel SNP. A panel of hap.03 HD subjects from Northern Europe (1 sample), Spain (4–6 samples), Portugal (4–6 samples), and Brazil (1 sample) were sequences, and representative sequencing analysis results are shown.
(PDF)

S9 Fig Allele-specific CRISPR/Cas9 target sites for Venezuelan HD subjects carrying mutant hap.03 haplotype. Fully reconstructed Venezuelan hap.03 sequences were compared to consensus allele of hap.08 allele from 1000 Genomes Project data (phase 3). Haplotype hap.08 represents the most common normal chromosome in Europeans and Americans (1000 Genomes Project, phase 3). A consensus sequence of hap.08 from the 1000 Genomes Project data represents the most frequent allele for a given site. Subsequently, we determined those PAM-altering SNPs that create and eliminate CRISPR/Cas9 PAM sites (NGG) in hap.03 and hap.08 haplotypes, respectively. In the region, 16 variations create NGG PAM sites only in hap.03 and therefore can be used for allele-specific CRISPR/Cas9 targeting aiming at silencing the mutant allele. Red arrows show an example where simultaneous application of CRISPR gRNAs is expected to selectively eliminate the transcription start site and expanded CAG repeat in the mutant *HTT*.
(PDF)

S10 Fig Distribution of residual age at onset in Venezuelan HD subjects. In order to generate phenotype data for GWA analysis aiming at discovering modifiers of HD subjects in Venezuela, a regression model described in Fig 1B was used to calculate the difference between observed age at onset and predicted age at onset based on CAG repeat size. Distributions of residual age at onset in Venezuelans were visually compared that in European HD subjects previously described. The mean and median of residual age at onset in Venezuelan HD were 0.368 and 0.403, respectively.
(PDF)

S11 Fig Quantile-quantile (QQ) plot. Levels of genomic inflation were assessed based on QQ plot. The inflation factor was 1.0197 by the median method.
(PDF)

S12 Fig Suggestive association signals on chromosomes 4 and 17. Suggestive modification signals on chromosome 4 (A) and 17 (B) are displayed. X-axis and Y-axis represent genomic coordinate (hg19) and -log10(p-value), respectively. Each grey circle is a SNP, and cyan trace represents recombination rates (secondary Y-axis) based on HapMap data. All exons were combined to represent a gene, in red or blue to represent genes on plus and minus strand, respectively.
(PDF)

S13 Fig Population-specific modification by rs12668183 on chromosome 7. Residual age at onset of HD subjects carrying 0, 1, or 2 minor alleles of the top SNP (i.e., rs12668183) are plotted for Venezuelan HD (A) and European HD subjects (B). Red horizontal lines represent mean residual age at onset.
(PDF)

S14 Fig Genetic modification score based on European HD subjects. Independent suggestive significant or higher SNPs in European HD subjects were used as a training set to construct the polygenic modification score of each HD subject. Briefly, for a given individual, effect sizes of SNPs were multiplied by the number of effect alleles to calculate the sum of the modification score. The same scoring method was applied to European HD subjects to estimate the maximum prediction power of the modification score (A). Next, the effect sizes and scoring SNPs based on European HD subjects were applied to calculate the genetic modification score of Venezuelan HD subjects and subsequently used to explain residual age at onset of Venezuelan HD subjects (B). For each HD population, residual age at onset was modeled as a function of genetic modification score to judge its significance.
(PDF)

S15 Fig Lack of contribution from CNV. (A) Focusing on the chromosome 7 region that generated genome-wide significant modification signals in Venezuelan HD, frequencies of CNVs are plotted over SNP association analysis results (grey circles). Black, blue, and red lines represent all CNVs, duplication, and deletion, respectively. Primary and secondary Y-axes represent SNP significances and frequencies of CNVs, respectively. X-axis represent genomic coordinate based on hg19 assembly.
(B) In order to judge the levels of contribution of CNV to significant modification signals in the chromosome 7 region, samples carrying CNVs were excluded for SNP association analysis in a mixed effect model. X-axis and Y-axis represent association analysis results using all samples (374 subjects) and those using samples w/o CNVs (317 subjects). Significance means -log10(p-value).
(PDF)

S16 Fig Equal contribution from the main family and other families. In order to determine whether the genome-wide significant modification signals were largely contributed by the main family, we split the data into the main family (218 subjects) and other families (156 subjects). Subsequently, statistical analysis was performed to determine whether residual age at onset in the main family was significantly different from that in the other families (A).
(B) Chromosome 7 top SNP (i.e., rs12668183) was also analyzed independently in the two groups to assess the significance in modification.
(PDF)

S17 Fig Extreme dichotomous analysis. As a confirmatory analysis of continuous phenotype data analysis, we performed extreme dichotomous analysis. Instead of using continuous residual age at onset data, we generated two groups representing the 10% extremes of age at onset. We then compared allele frequency of the markers between the extreme early onset group and the extreme late onset group. The X-axis represents continuous association analysis results using all samples, and Y-axis represents extreme dichotomous association analysis.
(PDF)

S18 Fig Potential genetic interaction with chromosome 7 modifier. We attempted to detect genetic interactions with chromosome 7 modifier locus. Venezuelan HD subjects were grouped by a chromosome 7 modifier SNP rs150393409; minor allele-carriers (A; 238 subjects) and non-carriers (B; 136 subjects). For each group, samples with top and bottom 40% residual age at onset were identified. Subsequently, GWA analysis was performed using dichotomized phenotype data in mixed effect model (see method section). Although not genome-wide significant, some regions in the genome suggested modification of HD in the presence (e.g., chromosome 8 in panel A) or absence (chromosome 12 in panel B) of chromosome 7 onset modifier.
(PDF)
