# Supplementary figures and images for: Population-specific genetic modification of Huntington's disease in Venezuela
Source: PLoS Genet. 2018 May 11;14(5):e1007274. doi: 10.1371/journal.pgen.1007274 (PMC5965898; doi:10.1371/journal.pgen.1007274)

S1 Fig

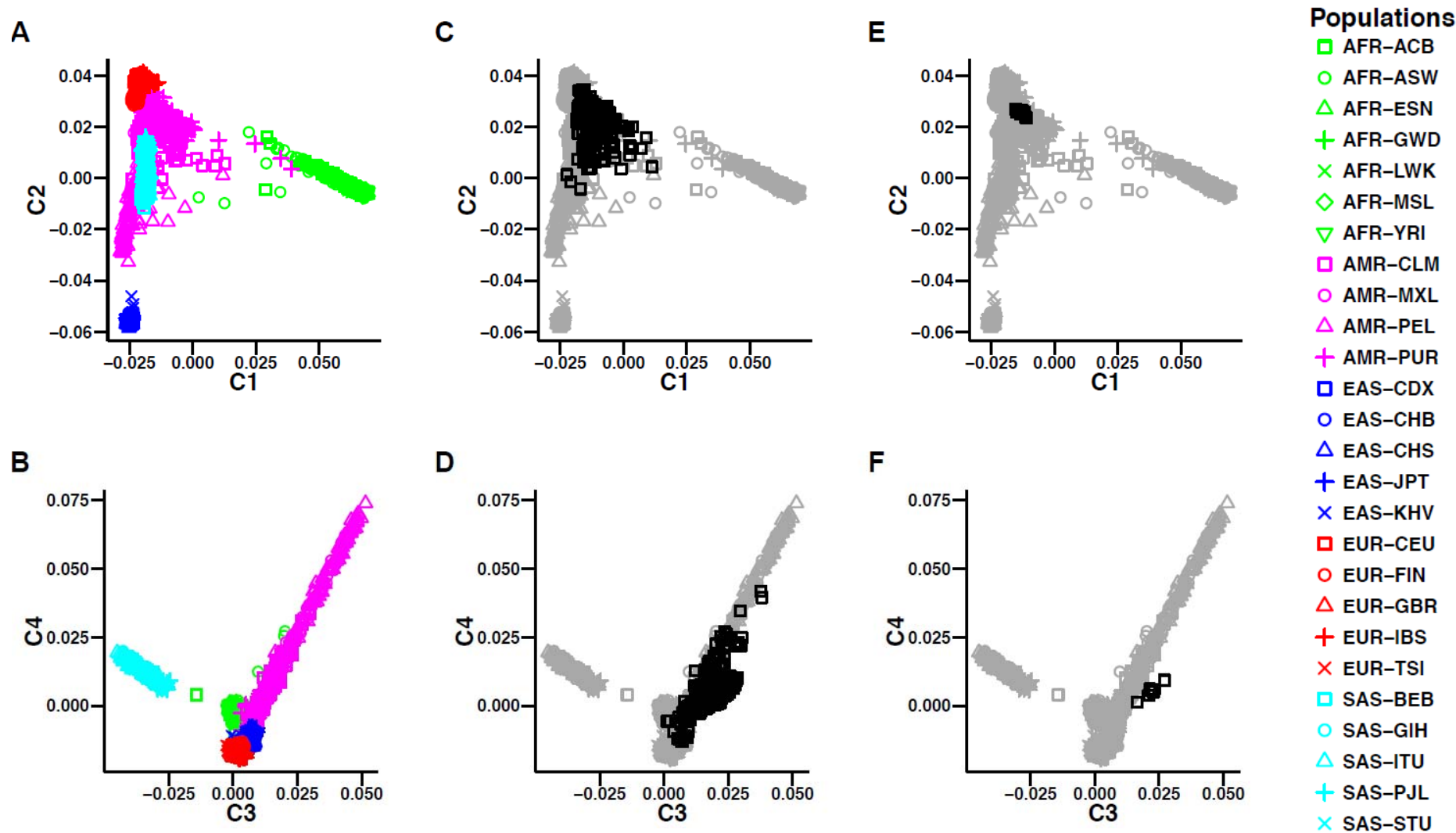

Supplement: S1 Fig — In order to confirm ancestry of Venezuelan HD study subjects, principal components analysis using genome-wide SNP data of samples was performed together with 1000 Genomes Project data (Phase 3; 2,504 samples from 26 populations). The first vs. second MDS (multidimensional scaling) values (top row), and the third vs. fourth MDS values (bottom row) were plotted. 1000 Genomes Project samples are represented by colored symbols (A and B), or open grey symbols (C, D, E, and F). Study subjects used for GWA analysis (C and D) or sequencing analysis (E and F) are indicated by open black squares. For detailed description of populations and symbols of 1000 Genomes Project, refer to http://www.internationalgenome.org/faq/which-populations-are-part-your-study/. (PDF) [file pgen.1007274.s001.pdf]

S2 Fig

**A**

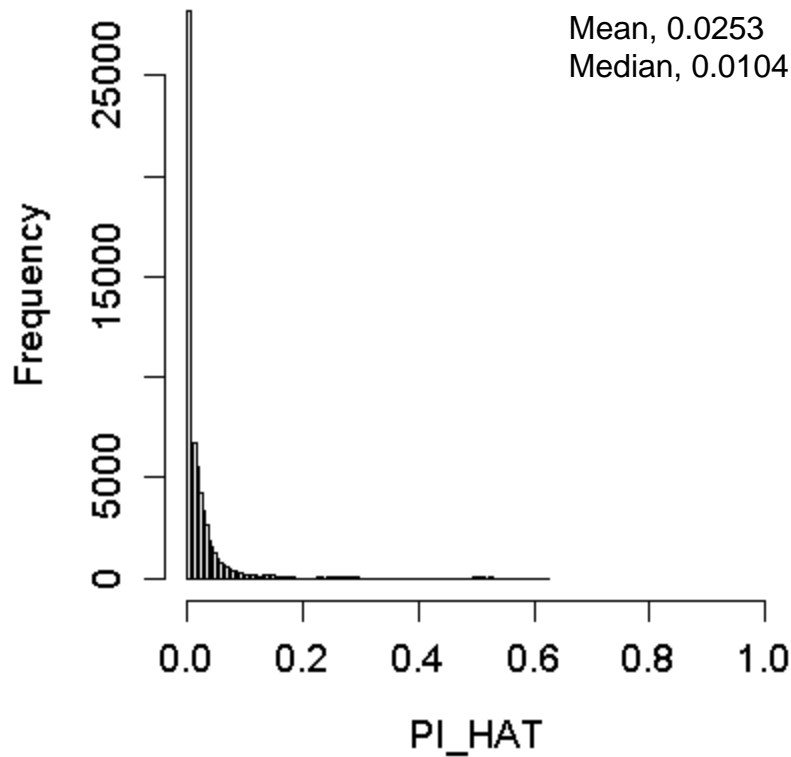

**B**

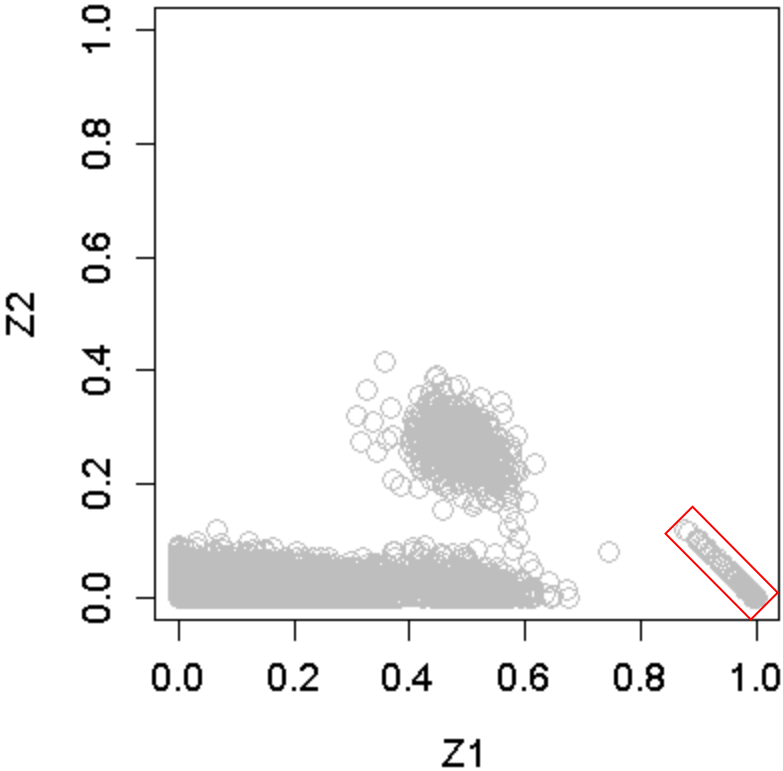

Supplement: S2 Fig — For QC-passed 374 Venezuelan HD study subjects analyzed in GWA analysis, familial relationships were inferred based on genome-wide genotype data. Pair-wise IBD (identity-by-descent) values were estimated by the PLINK program. From 374 unique study subjects, IBD values were estimated for 69,751 meaningful pairs. The mean of PI_HAT value (indicative of IBD) was 0.0253 (A). Based on Z1 and Z2 values from the PLINK IBD estimation, we identified 170 unique parent-child pairs comprising 216 subjects (B, red rectangle). In addition, siblings and other relationships were also revealed (B). (PDF) [file pgen.1007274.s002.pdf]

S3 Fig

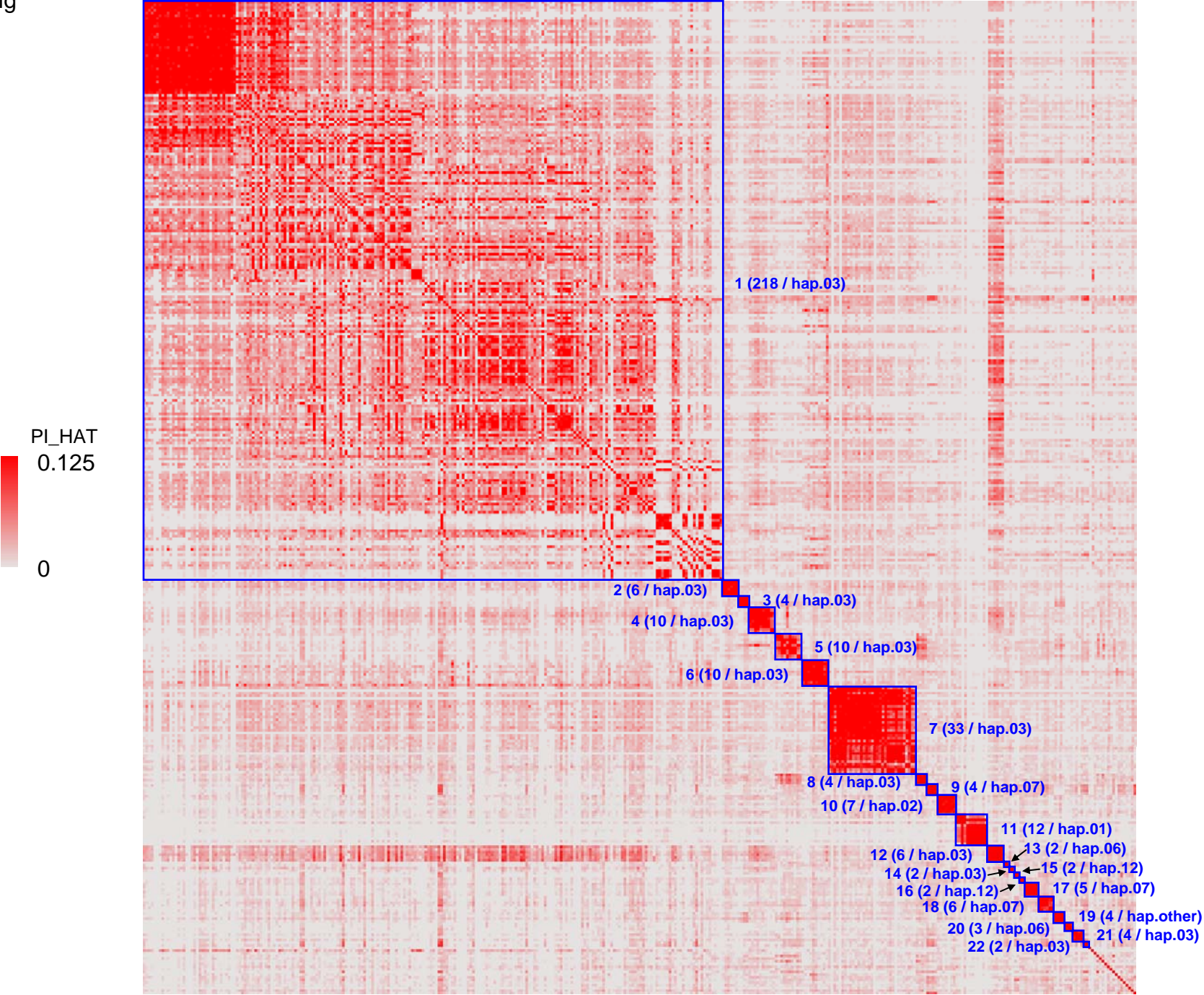

Supplement: S3 Fig — Based on pair-wise IBD estimations, families were reconstructed. A pair of related individuals whose PLINK PI_HAT value was greater than 0.125 (representing first cousin or closer relationships) were combined, and this process was repeated exhaustively until all related individuals were combined, generating 22 families with variable sizes. Therefore, an individual within a given re-constructed family has at least one relative. Families were numbered arbitrarily and were separated by blue boundary line. The size of each family is provided in a parenthesis, and haplotype of HD disease chromosome is indicated in parenthesis after slash. Unrelated individuals (18) are at the bottom-right of the plot without family name. (PDF) [file pgen.1007274.s003.pdf]

S4 Fig

**A**

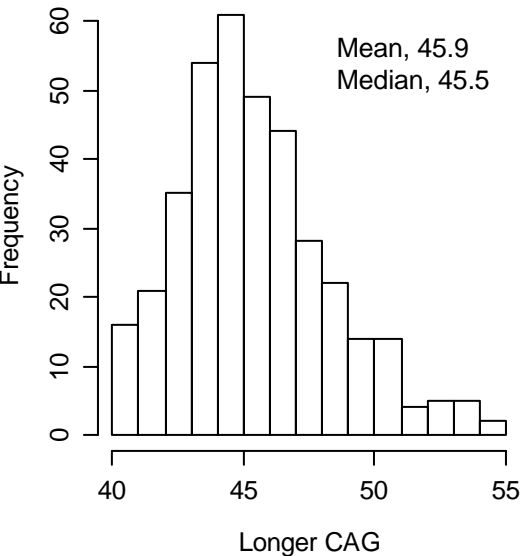

**B**

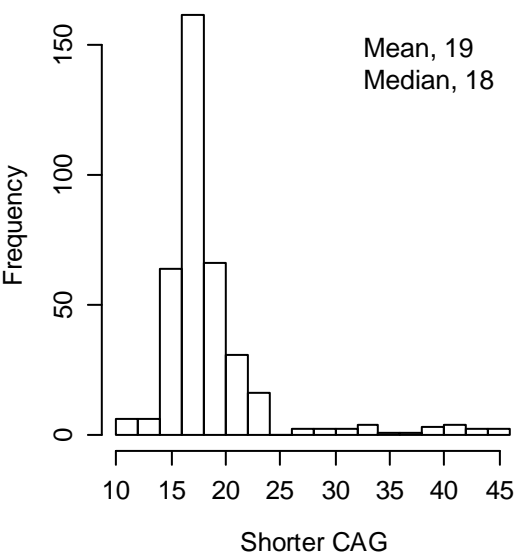

**C**

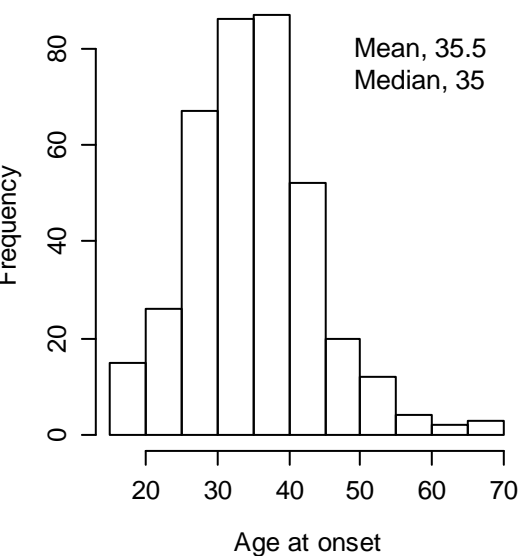

Supplement: S4 Fig — Following sample QC analysis, 374 Venezuelan HD subjects including 12 homozygote HD individuals were analyzed. Histogram plots show distributions of longer CAG repeats (A), shorter CAG repeats (B), and age at onset of motor symptoms (C). The means of longer CAG, shorter CAG, and age at onset of motor symptoms are 45.9 (A), 19 (B), and 35.5 (C), respectively. (PDF) [file pgen.1007274.s004.pdf]

S5 Fig

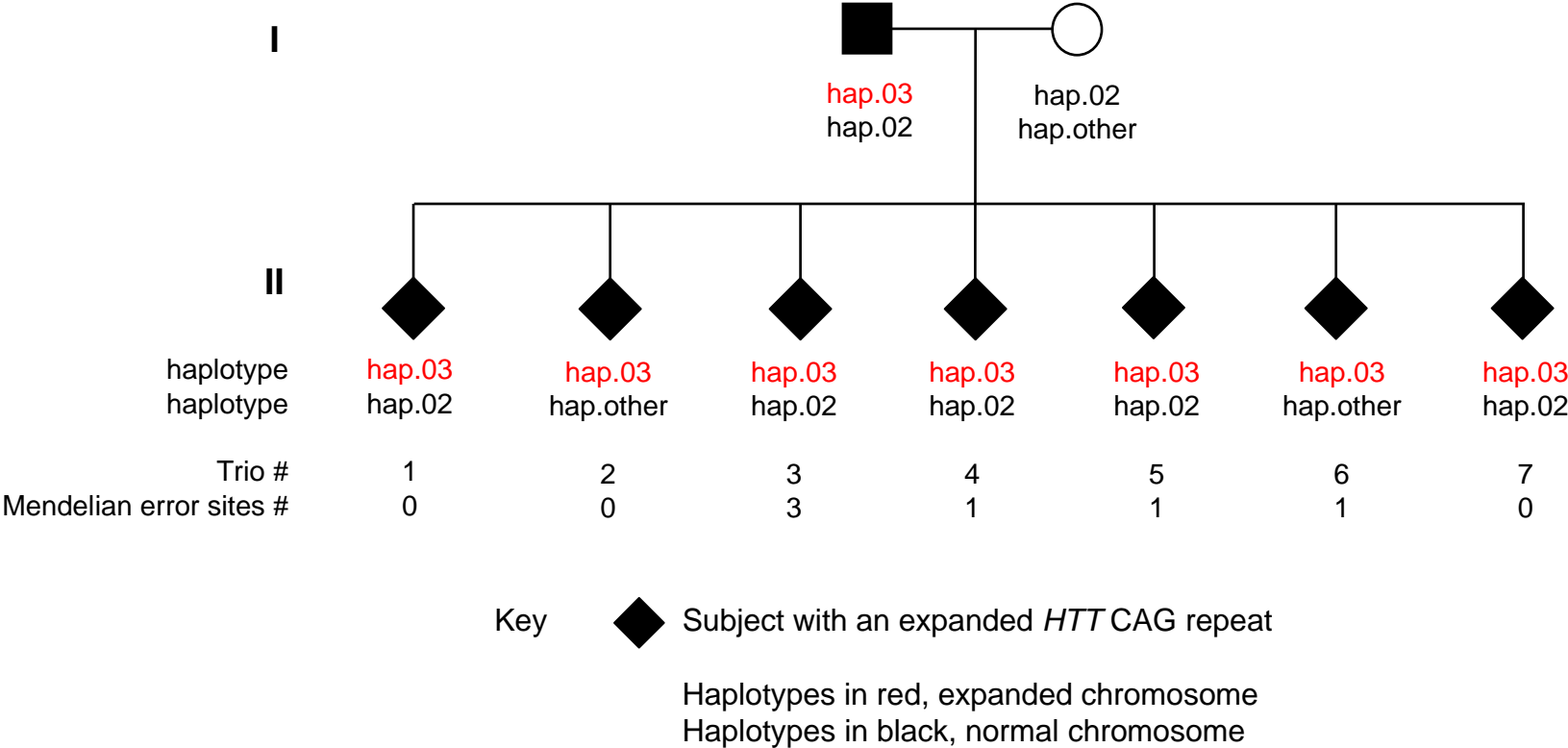

Supplement: S5 Fig — Nine subjects including one non-HD parent were used for whole genome sequencing. This family inherits the hap.03 haplotype on the mutant chromosome as the majority of Venezuelan HD subjects do. For each subject, haplotypes of mutant chromosome (red) and normal chromosome (black) are indicated. In addition, to determine the phased sequence of HTT in this family, we performed trio phasing for each trio comprising parents and a child. As indicated by "Trio #", 7 trios were used for phasing analysis. For each trio phasing a small number of Mendelian errors were detected. Diamond symbols were used for children to maintain confidentiality. (PDF) [file pgen.1007274.s005.pdf]

S7 Fig

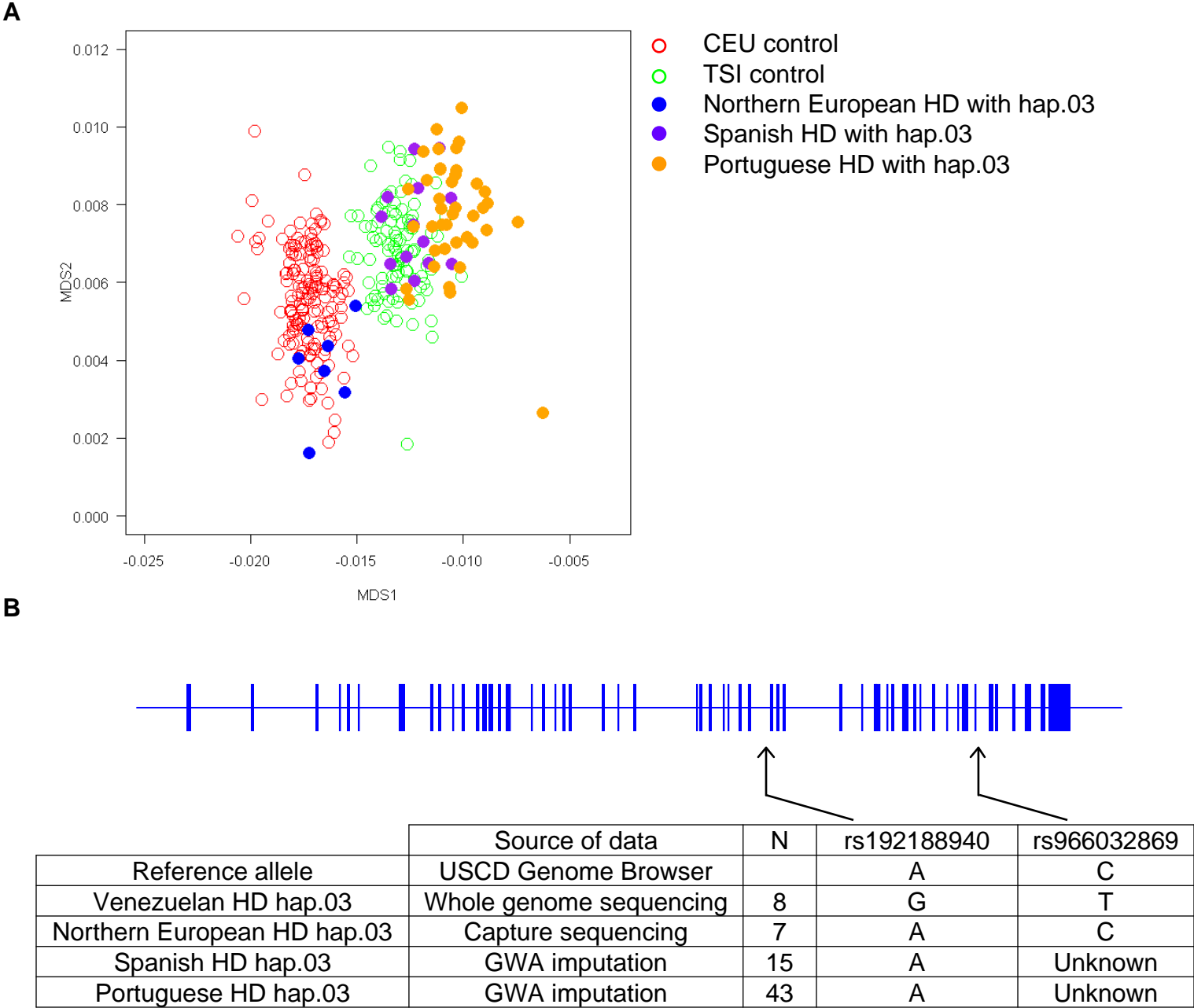

Supplement: S7 Fig — (A) Northern (filled blue circles) or Southern European HD subjects (filled purple and orange circles representing Spanish and Portuguese HD, respectively) carrying hap.03 as the mutant chromosome were identified using phased haplotypes of GWA data. A scatter plot shows MDS 1 and 2 values from principal components analysis of GWA data relative to control populations such as CEU and TSI. (B) Consensus alleles of the hap.03 haplotype in Venezuelan HD were obtained from trio phasing of family samples. In addition, consensus alleles of the hap.03 disease haplotype in 7 independent Northern European HD subjects were obtained from capture sequencing analysis (7 samples). Direct comparisons of consensus alleles revealed 2 sites (including a novel SNP) where alleles are different between Venezuelan and European hap.03 disease haplotypes. For the known SNP site (rs192188940), imputed GWA data for Spanish (15 samples) and Portuguese HD (43 samples) with hap.03 haplotype are also indicated. Since the novel SNP is not part of the 1000 Genomes Project reference panel, rs966032869 was not imputed in GWA data. (PDF) [file pgen.1007274.s007.pdf]

S8 Fig

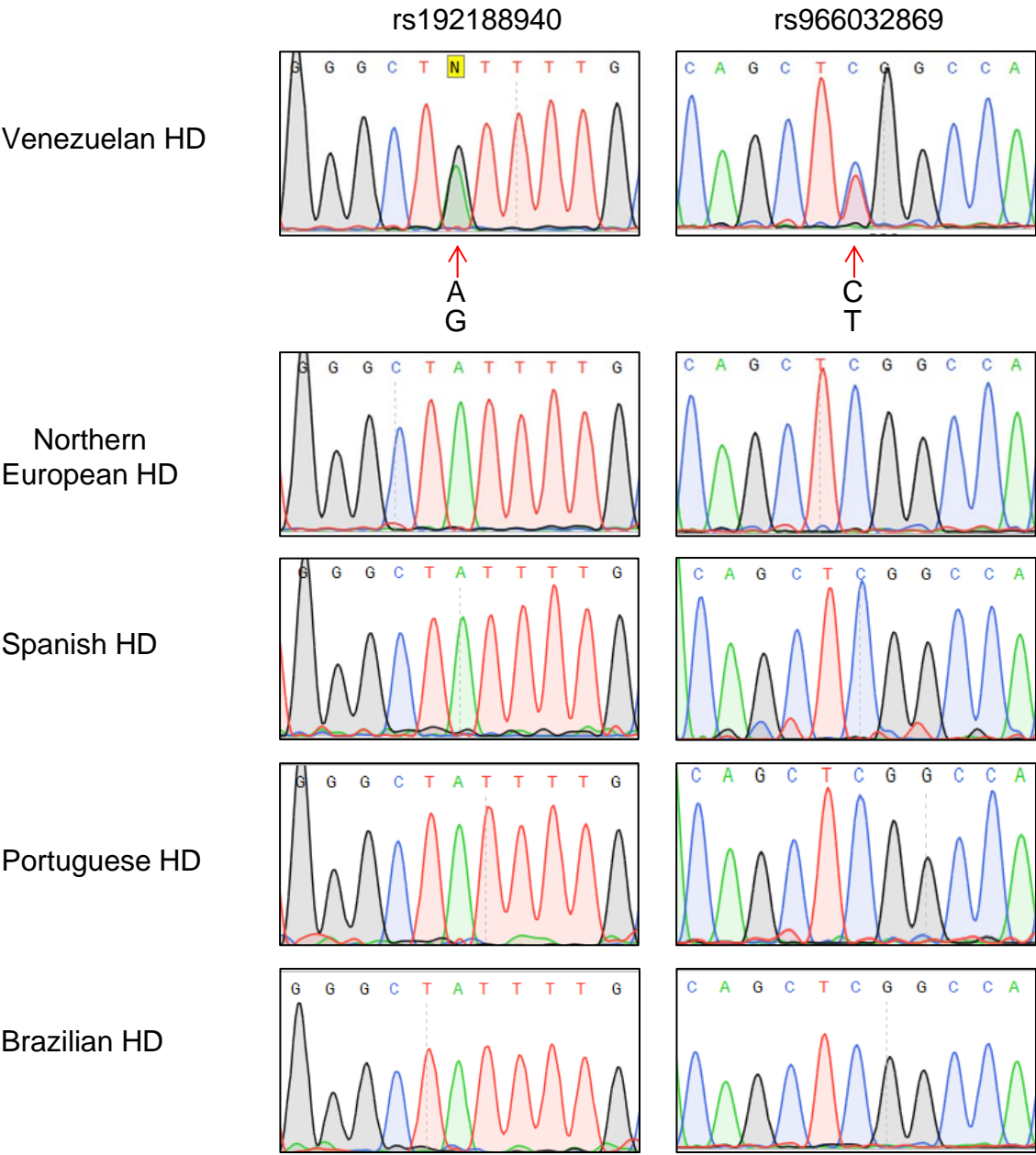

Supplement: S8 Fig — Sanger sequencing analysis was performed to validate the genotypes of Venezuelan hap.03-specific SNPs including a novel SNP. A panel of hap.03 HD subjects from Northern Europe (1 sample), Spain (4–6 samples), Portugal (4–6 samples), and Brazil (1 sample) were sequences, and representative sequencing analysis results are shown. (PDF) [file pgen.1007274.s008.pdf]

S9 Fig

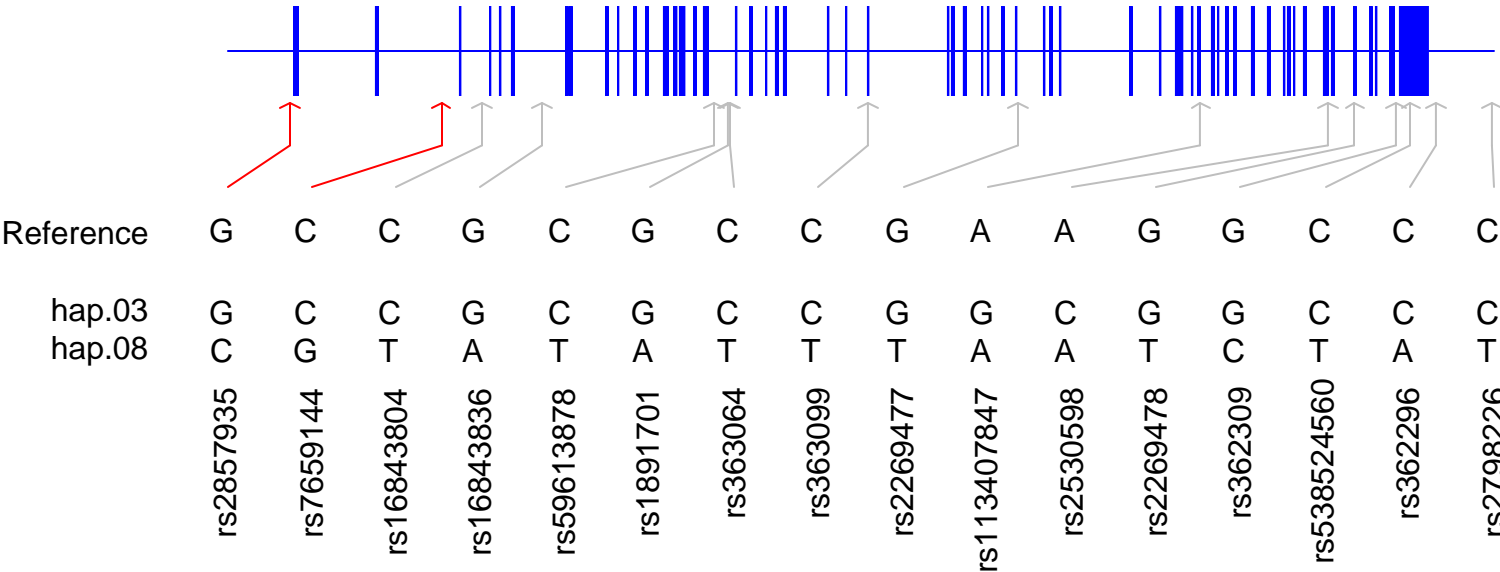

Supplement: S9 Fig — Fully reconstructed Venezuelan hap.03 sequences were compared to consensus allele of hap.08 allele from 1000 Genomes Project data (phase 3). Haplotype hap.08 represents the most common normal chromosome in Europeans and Americans (1000 Genomes Project, phase 3). A consensus sequence of hap.08 from the 1000 Genomes Project data represents the most frequent allele for a given site. Subsequently, we determined those PAM-altering SNPs that create and eliminate CRISPR/Cas9 PAM sites (NGG) in hap.03 and hap.08 haplotypes, respectively. In the region, 16 variations create NGG PAM sites only in hap.03 and therefore can be used for allele-specific CRISPR/Cas9 targeting aiming at silencing the mutant allele. Red arrows show an example where simultaneous application of CRISPR gRNAs is expected to selectively eliminate the transcription start site and expanded CAG repeat in the mutant HTT. (PDF) [file pgen.1007274.s009.pdf]

S10 Fig

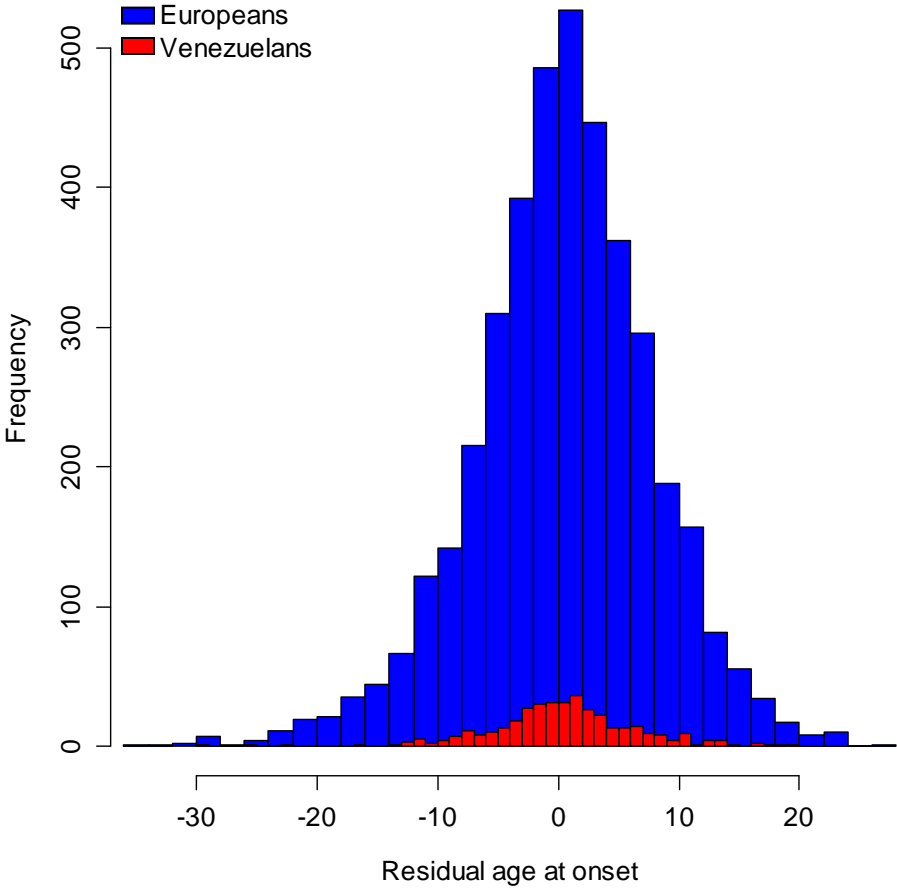

Supplement: S10 Fig — In order to generate phenotype data for GWA analysis aiming at discovering modifiers of HD subjects in Venezuela, a regression model described in Fig 1B was used to calculate the difference between observed age at onset and predicted age at onset based on CAG repeat size. Distributions of residual age at onset in Venezuelans were visually compared that in European HD subjects previously described. The mean and median of residual age at onset in Venezuelan HD were 0.368 and 0.403, respectively. (PDF) [file pgen.1007274.s010.pdf]

S11 Fig

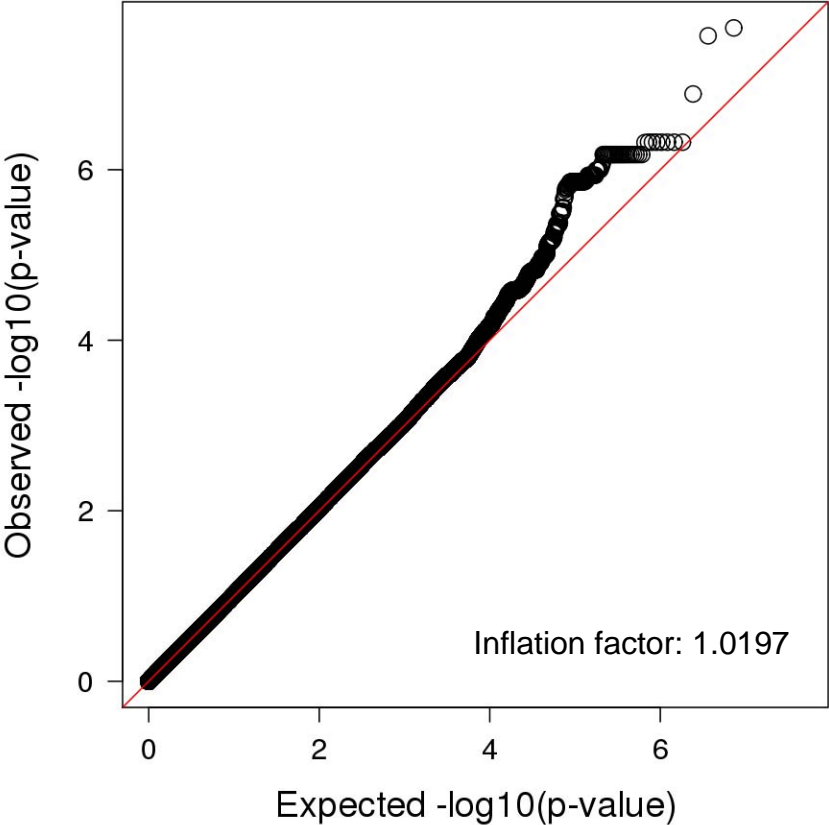

Supplement: S11 Fig — Levels of genomic inflation were assessed based on QQ plot. The inflation factor was 1.0197 by the median method. (PDF) [file pgen.1007274.s011.pdf]

S12 Fig

**A**

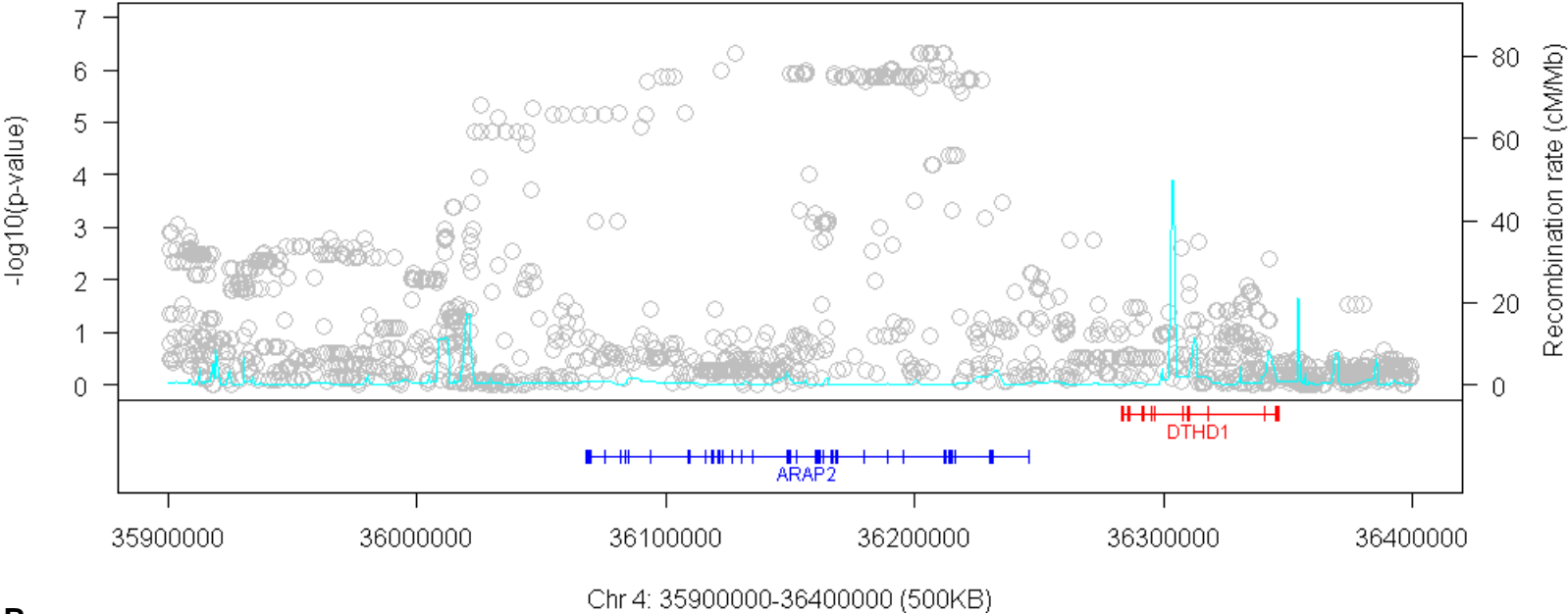

**B**

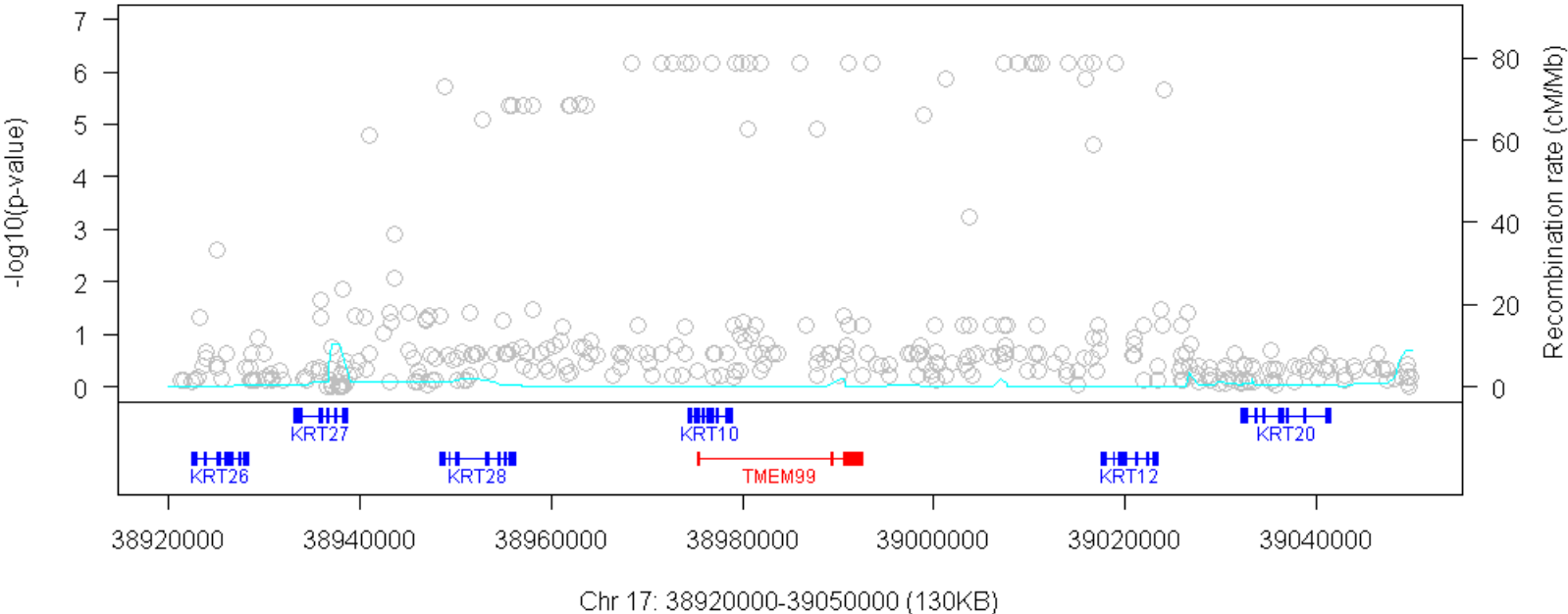

Supplement: S12 Fig — Suggestive modification signals on chromosome 4 (A) and 17 (B) are displayed. X-axis and Y-axis represent genomic coordinate (hg19) and -log10(p-value), respectively. Each grey circle is a SNP, and cyan trace represents recombination rates (secondary Y-axis) based on HapMap data. All exons were combined to represent a gene, in red or blue to represent genes on plus and minus strand, respectively. (PDF) [file pgen.1007274.s012.pdf]

S13 Fig

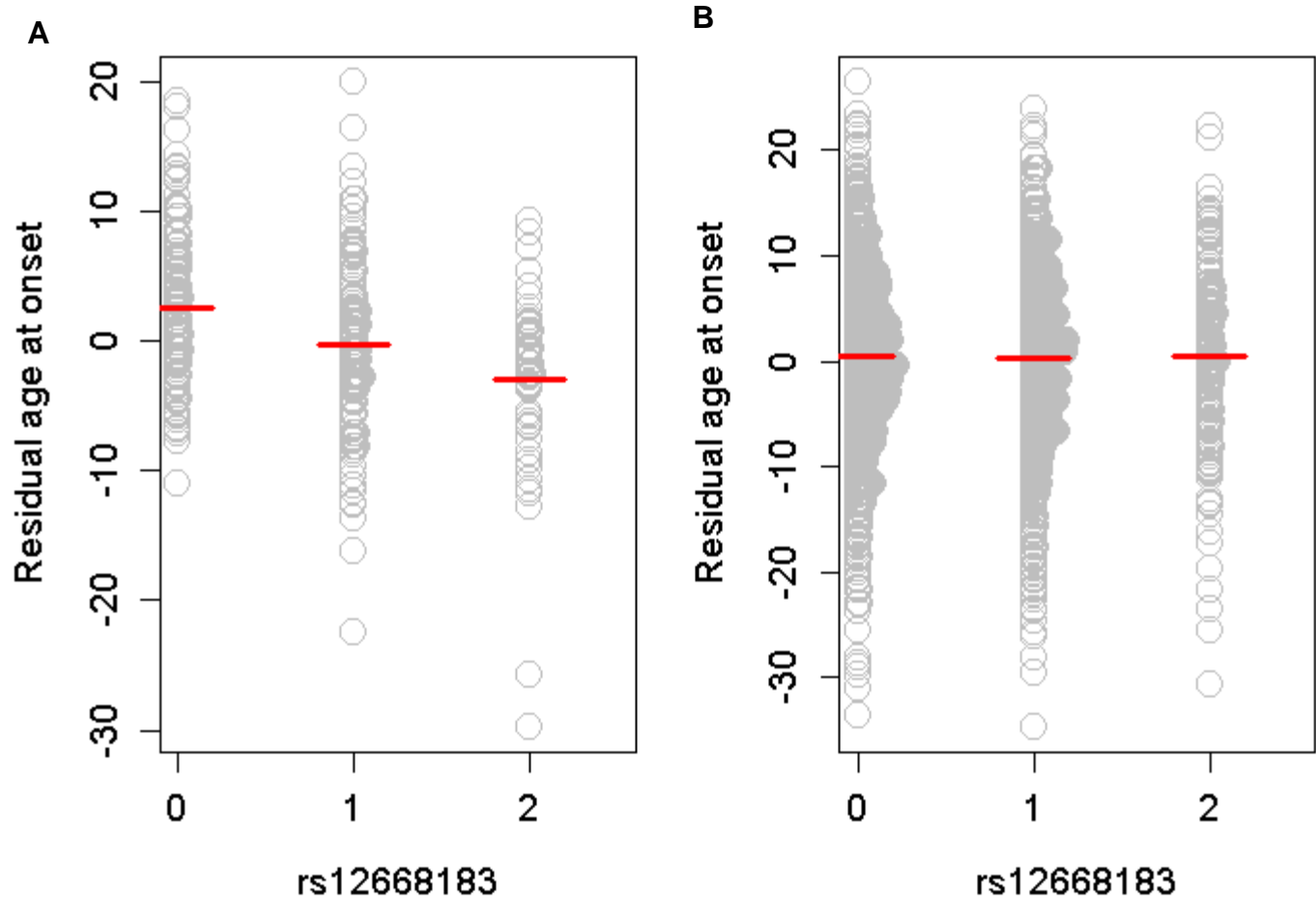

Supplement: S13 Fig — Residual age at onset of HD subjects carrying 0, 1, or 2 minor alleles of the top SNP (i.e., rs12668183) are plotted for Venezuelan HD (A) and European HD subjects (B). Red horizontal lines represent mean residual age at onset. (PDF) [file pgen.1007274.s013.pdf]

S14 Fig

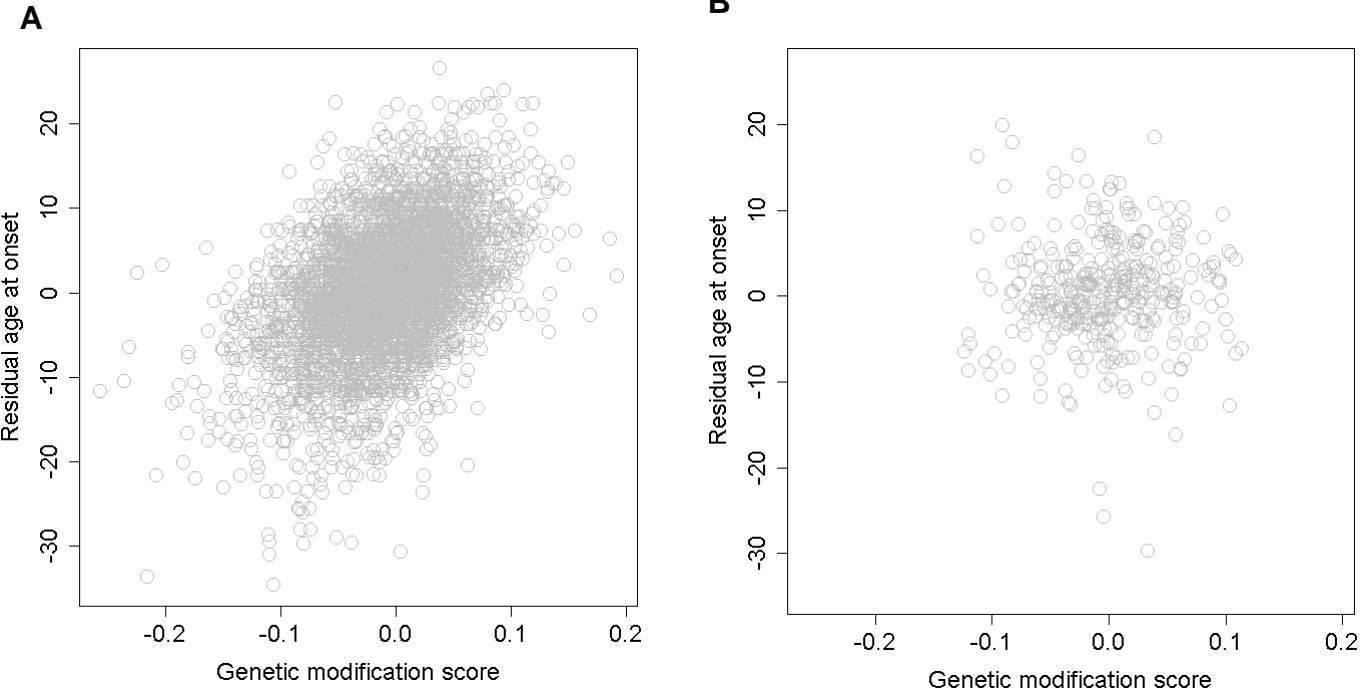

Supplement: S14 Fig — Independent suggestive significant or higher SNPs in European HD subjects were used as a training set to construct the polygenic modification score of each HD subject. Briefly, for a given individual, effect sizes of SNPs were multiplied by the number of effect alleles to calculate the sum of the modification score. The same scoring method was applied to European HD subjects to estimate the maximum prediction power of the modification score (A). Next, the effect sizes and scoring SNPs based on European HD subjects were applied to calculate the genetic modification score of Venezuelan HD subjects and subsequently used to explain residual age at onset of Venezuelan HD subjects (B). For each HD population, residual age at onset was modeled as a function of genetic modification score to judge its significance. (PDF) [file pgen.1007274.s014.pdf]

S15 Fig

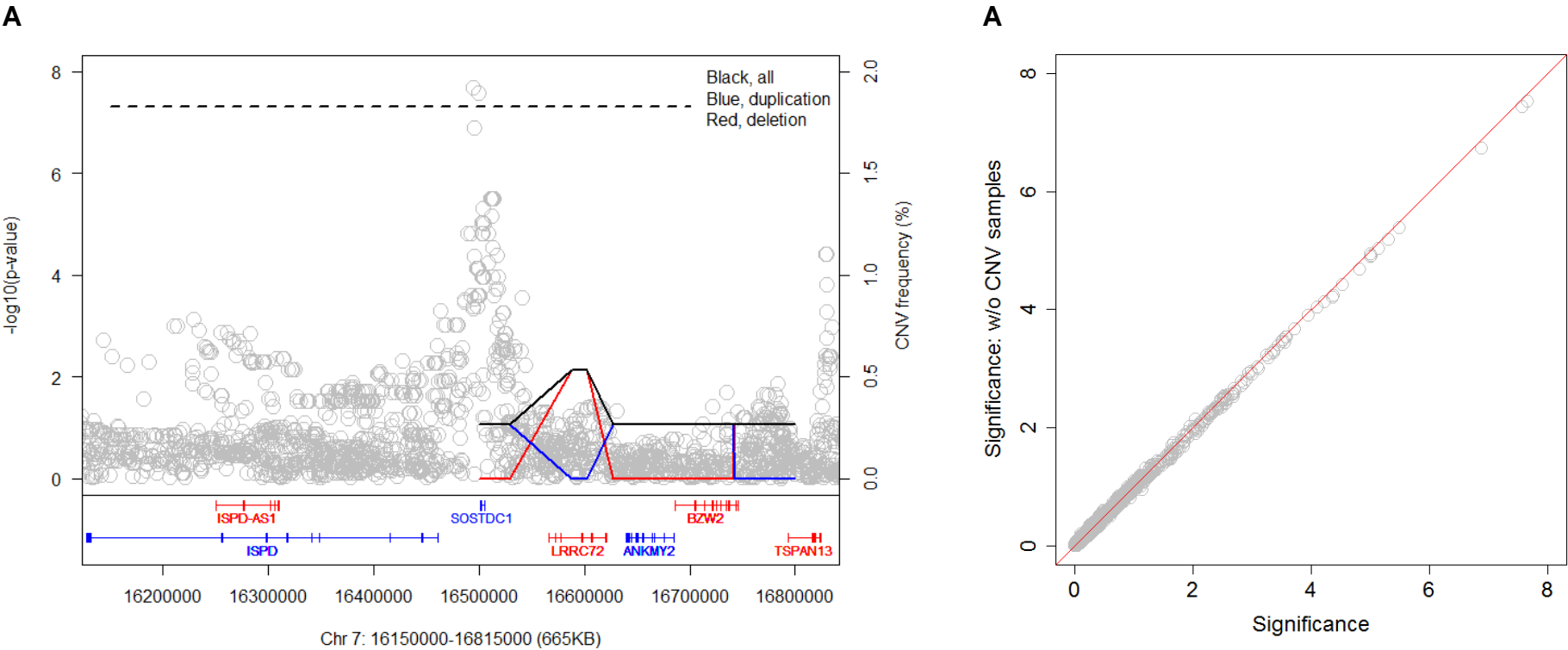

Supplement: S15 Fig — (A) Focusing on the chromosome 7 region that generated genome-wide significant modification signals in Venezuelan HD, frequencies of CNVs are plotted over SNP association analysis results (grey circles). Black, blue, and red lines represent all CNVs, duplication, and deletion, respectively. Primary and secondary Y-axes represent SNP significances and frequencies of CNVs, respectively. X-axis represent genomic coordinate based on hg19 assembly. (B) In order to judge the levels of contribution of CNV to significant modification signals in the chromosome 7 region, samples carrying CNVs were excluded for SNP association analysis in a mixed effect model. X-axis and Y-axis represent association analysis results using all samples (374 subjects) and those using samples w/o CNVs (317 subjects). Significance means -log10(p-value). (PDF) [file pgen.1007274.s015.pdf]

S17 Fig

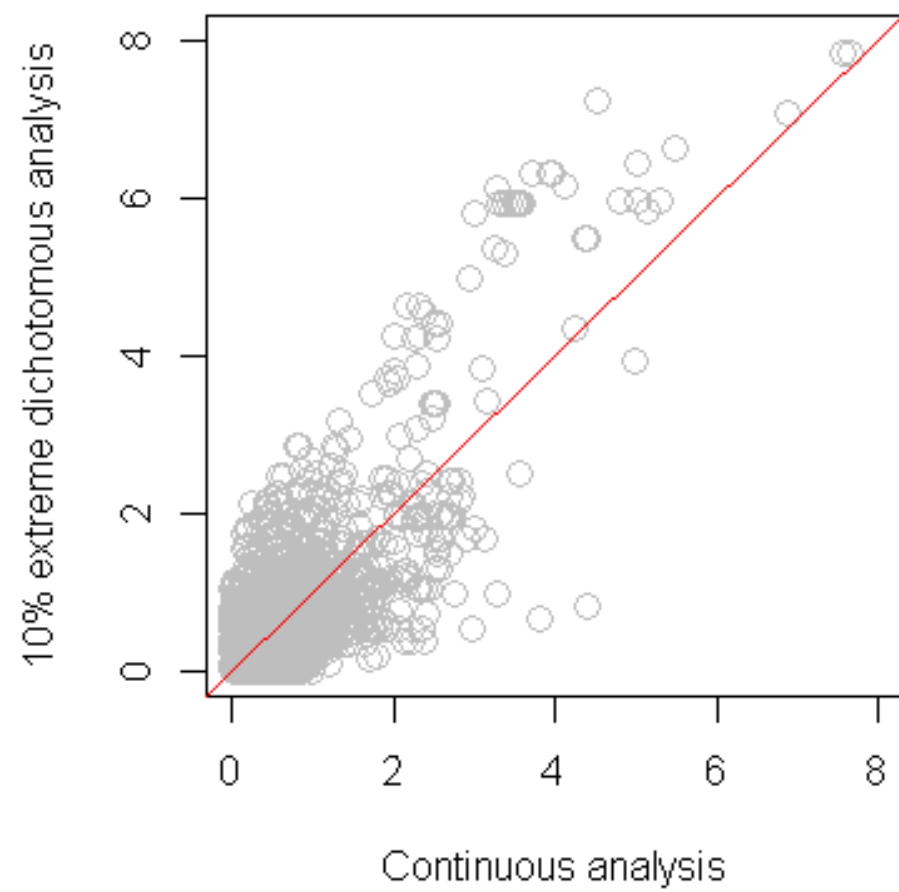

Supplement: S17 Fig — As a confirmatory analysis of continuous phenotype data analysis, we performed extreme dichotomous analysis. Instead of using continuous residual age at onset data, we generated two groups representing the 10% extremes of age at onset. We then compared allele frequency of the markers between the extreme early onset group and the extreme late onset group. The X-axis represents continuous association analysis results using all samples, and Y-axis represents extreme dichotomous association analysis. (PDF) [file pgen.1007274.s017.pdf]

S18 Fig

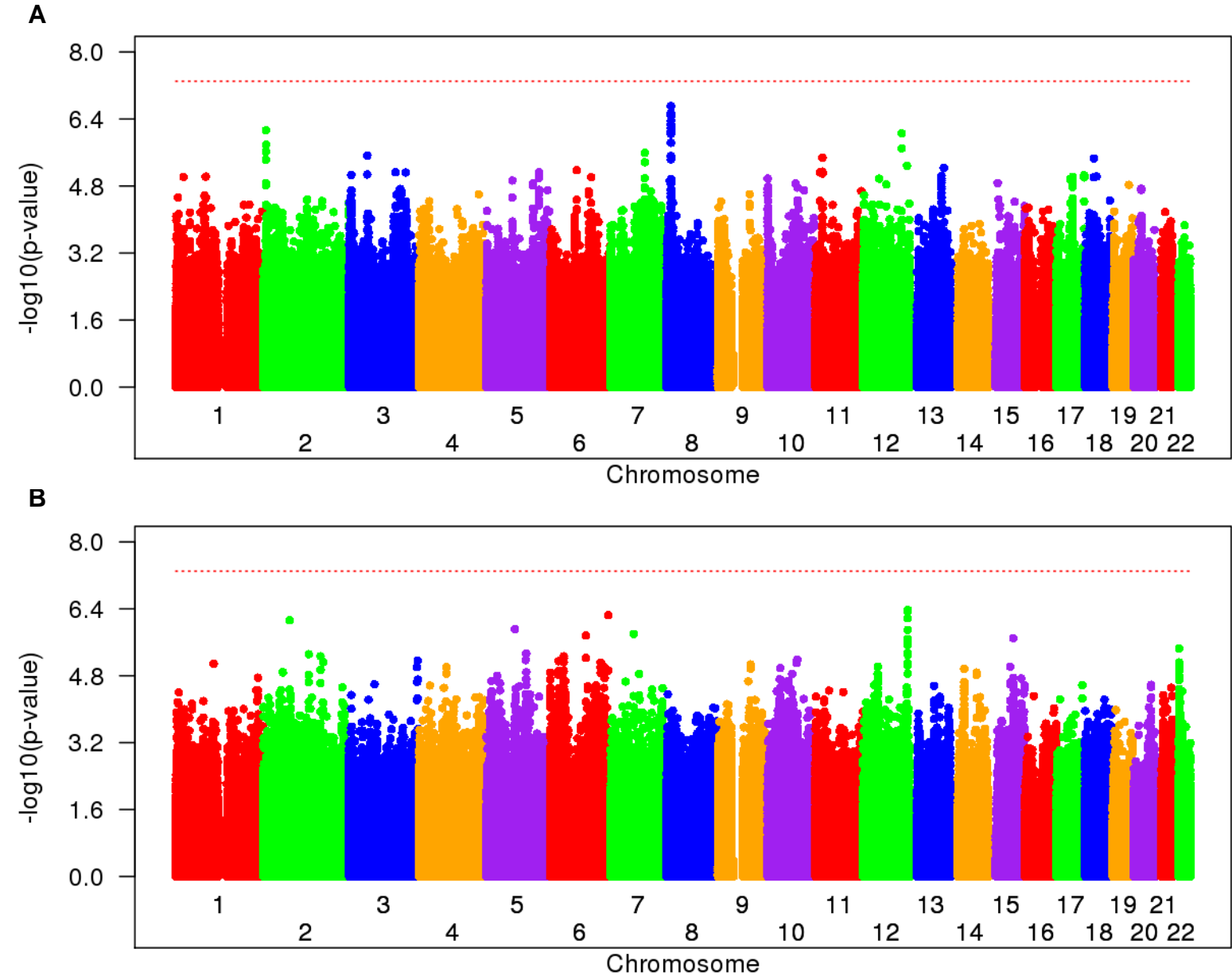

Supplement: S18 Fig — We attempted to detect genetic interactions with chromosome 7 modifier locus. Venezuelan HD subjects were grouped by a chromosome 7 modifier SNP rs150393409; minor allele-carriers (A; 238 subjects) and non-carriers (B; 136 subjects). For each group, samples with top and bottom 40% residual age at onset were identified. Subsequently, GWA analysis was performed using dichotomized phenotype data in mixed effect model (see method section). Although not genome-wide significant, some regions in the genome suggested modification of HD in the presence (e.g., chromosome 8 in panel A) or absence (chromosome 12 in panel B) of chromosome 7 onset modifier. (PDF) [file pgen.1007274.s018.pdf]
