# Supplementary material for: Population-specific genetic modification of Huntington's disease in Venezuela
Source: PLoS Genet. 2018 May 11;14(5):e1007274. doi: 10.1371/journal.pgen.1007274 (PMC5965898; doi:10.1371/journal.pgen.1007274)
Supplement: S6 Fig — (A) For each trio, trivial phasing analysis was performed using the BEAGLE program, and 7 phased mutant chromosomes were merged to identify consensus alleles of hap.03 disease haplotype in this family. We detected 6 unique Mendelian error sites, 3,231 unphaseable sites due to missing data, and 7 inconsistent sites. In total 99.02% of bases were determined. Examples of inconsistent sites are shown in panel B. N and D represent missing and deletion. (PDF) [file pgen.1007274.s006.pdf]

S6 Fig

A

Number of trios	Total sites	All missing sites	Mendelian error sites	Unphaseable sites	Inconsistent sites	Phased sites (%)
7	189,280	179	6	3231	7	187429 (99.02)

B

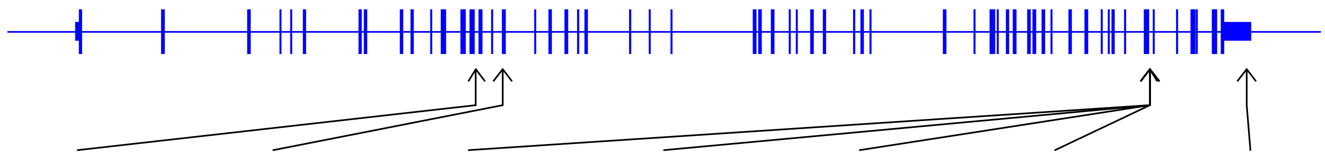

Trio 1	C	G	A	C	A	C	C
Trio 2	N	G	A	C	A	C	N
Trio 3	C	G	A	C	D	D	C
Trio 4	T	A	A	D	D	C	N
Trio 5	T	A	A	C	A	C	T
Trio 6	N	A	A	D	D	C	C
Trio 7	C	G	D	D	D	C	C
	3133911	3137674	3231045	3231046	3231047	3231048	3245057
