# Supplementary material for: Population-specific genetic modification of Huntington's disease in Venezuela
Source: PLoS Genet. 2018 May 11;14(5):e1007274. doi: 10.1371/journal.pgen.1007274 (PMC5965898; doi:10.1371/journal.pgen.1007274)
Supplement: S16 Fig — In order to determine whether the genome-wide significant modification signals were largely contributed by the main family, we split the data into the main family (218 subjects) and other families (156 subjects). Subsequently, statistical analysis was performed to determine whether residual age at onset in the main family was significantly different from that in the other families (A). (B) Chromosome 7 top SNP (i.e., rs12668183) was also analyzed independently in the two groups to assess the significance in modification. (PDF) [file pgen.1007274.s016.pdf]

S16 Fig

A

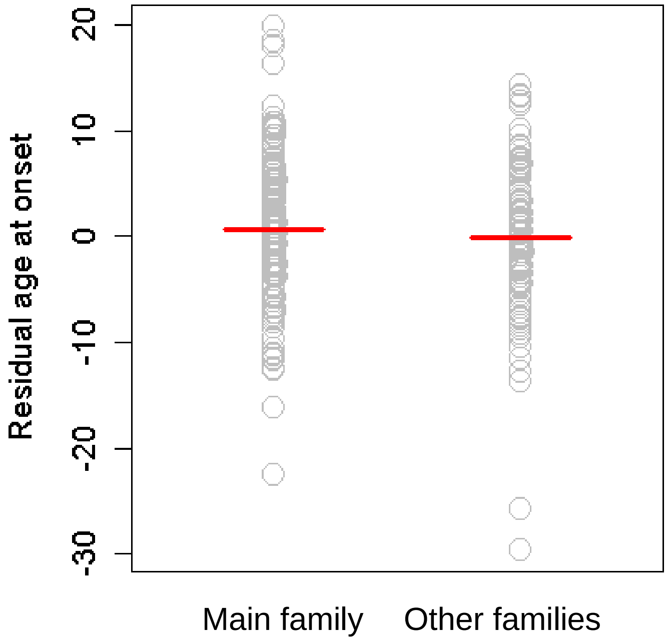

(Wilcoxon test p-value, 0.389)

B

rs12668183	Effect size (years / minor allele)	P-value
All samples (n=374)	-2.76886	2.17E-08
Main family (n=218)	-2.50069	6.82E-05
Other families (n = 156)	-3.5061	1.70E-05
